# PknG senses amino acid availability to control metabolism and virulence of *Mycobacterium tuberculosis*

**DOI:** 10.1371/journal.ppat.1006399

**Published:** 2017-05-17

**Authors:** Barbara Rieck, Giulia Degiacomi, Michael Zimmermann, Alessandro Cascioferro, Francesca Boldrin, Natalie R. Lazar-Adler, Andrew R. Bottrill, Fabien le Chevalier, Wafa Frigui, Marco Bellinzoni, María-Natalia Lisa, Pedro M. Alzari, Liem Nguyen, Roland Brosch, Uwe Sauer, Riccardo Manganelli, Helen M. O’Hare

**Affiliations:** 1 Department of Infection, Immunity and Inflammation, University of Leicester, Leicester, United Kingdom; 2 Department of Molecular Medicine, University of Padua, Padova, Italy; 3 Institute of Molecular Systems Biology, ETH Zurich, Zurich, Switzerland; 4 Institut Pasteur, Integrated Mycobacterial Pathogenomics Unit, Paris, France; 5 Core Biotechnology Services, University of Leicester, Leicester, United Kingdom; 6 Institut Pasteur, Unité de Microbiologie Structurale and CNRS-UMR3528, Paris, France; 7 Department of Molecular Biology and Microbiology, Case Western Reserve University School of Medicine, Cleveland, Ohio, United States of America; 8 Department of Molecular and Cellular Bioscience, University of Leicester, Leicester, United Kingdom; National Institutes of Health, UNITED STATES

## Abstract

Sensing and response to changes in nutrient availability are essential for the lifestyle of environmental and pathogenic bacteria. Serine/threonine protein kinase G (PknG) is required for virulence of the human pathogen *Mycobacterium tuberculosis*, and its putative substrate GarA regulates the tricarboxylic acid cycle in *M*. *tuberculosis* and other Actinobacteria by protein-protein binding. We sought to understand the stimuli that lead to phosphorylation of GarA, and the roles of this regulatory system in pathogenic and non-pathogenic bacteria. We discovered that *M*. *tuberculosis* lacking *garA* was severely attenuated in mice and macrophages and furthermore that GarA lacking phosphorylation sites failed to restore the growth of *garA* deficient *M*. *tuberculosis* in macrophages. Additionally we examined the impact of genetic disruption of *pknG* or *garA* upon protein phosphorylation, nutrient utilization and the intracellular metabolome. We found that phosphorylation of GarA requires PknG and depends on nutrient availability, with glutamate and aspartate being the main stimuli. Disruption of *pknG* or *garA* caused opposing effects on metabolism: a defect in glutamate catabolism or depletion of intracellular glutamate, respectively. Strikingly, disruption of the phosphorylation sites of GarA was sufficient to recapitulate defects caused by *pknG* deletion. The results suggest that GarA is a cellular target of PknG and the metabolomics data demonstrate that the function of this signaling system is in metabolic regulation. This function in amino acid homeostasis is conserved amongst the Actinobacteria and provides an example of the close relationship between metabolism and virulence.

## Author summary

A key feature of the pathogen *Mycobacterium tuberculosis* is its ability to survive and replicate within human macrophages. Protein kinase G (PknG) is known to be required for virulence of *M*. *tuberculosis* and is the only bacterial serine/threonine protein kinase to be known as a virulence factor. However, the molecular mechanisms underlying its function in virulence are unknown and the role(s) of PknG are controversial. Here, we disrupted the genes encoding PknG and its putative substrate GarA in *M*. *tuberculosis* and related non-pathogenic *Mycobacterium smegmatis*. We observed changes in protein phosphorylation that suggest GarA is the substrate of PknG, and changes in growth and metabolome that establish this pair of proteins as a bone fide system for metabolic regulation. We also observed a dramatic impact on the ability of GarA-deficient *M*. *tuberculosis* to grow and survive in macrophages and mice. This highlights the link between metabolism and virulence and suggests that *M*. *tuberculosis* inside macrophages may have restricted access to amino acids. Our study also provides a first indication of the nutrients that may be sensed by *M*. *tuberculosis* inside macrophages and provides new insights into the rate and reversibility of serine/threonine phosphorylation in bacteria.

## Introduction

*Mycobacterium tuberculosis* is the causative agent of TB, and remains one of the world’s biggest health threats. Existing vaccination and drug treatment regimens may be circumvented by *M*. *tuberculosis* through sophisticated adaptation and resistance mechanisms. New insights into the regulatory and signal transduction networks and metabolism of *M*. *tuberculosis* are needed to better understand the biology of this outstanding pathogen. Genome analyses revealed that *M*. *tuberculosis* encodes 11 serine/threonine protein kinases (STPKs), some of which play essential roles for viability or virulence [1,2]. There is wide interest in these kinases as routes to understand the virulence strategies of *M*. *tuberculosis* and as potential therapeutic targets [3]. For these reasons, *M*. *tuberculosis* has become a target organism for research into the general mechanisms of signaling by serine and threonine phosphorylation in bacteria [2, 4].

PknG has been the focus of a number of studies because of its essentiality for virulence [5, 6] and its involvement in regulating industrial glutamate production by *Corynebacterium glutamicum* [7]. The kinase substrate GarA is also essential in *M*. *tuberculosis* and has been strongly associated to metabolic regulation [8, 9]. GarA controls the activities of three enzymes linking glutamate metabolism to the TCA cycle, and genetic disruption of *garA* leads to a distinctive nutrient-dependent phenotype in fast-growing non-pathogenic *Mycobacterium smegmatis* [9]. Although there is strong genetic evidence for the requirement of PknG for virulence, multiple alternative mechanisms have been proposed [10–12]. A major challenge for this and other bacterial STPKs is to determine the mechanisms underlying observed genetic essentiality, and to move beyond study of proteins *in vitro* to determine the physiological substrates and functions of kinases. We have previously presented evidence that phosphorylation of GarA switches off its regulatory functions, and that phosphorylated GarA can be found in *M*. *tuberculosis* and *M*. *smegmatis* [8]. However, the molecular or environmental signals that trigger GarA phosphorylation were unknown and the role of PknG in regulating metabolism (via GarA) has been controversial. Initially PknG was thought to inhibit phagosome-lysosome fusion [6], and *pknG* disruption in *Mycobacterium bovis* BCG caused no growth defect [13], raising the possibility that PknG played different roles in pathogenic *Mycobacterium* spp compared to non-pathogens [14, 15]. Subsequent research has shown that *pknG* mutants in different *Mycobacterium* spp. have increased antibiotic sensitivity and reduced biofilm formation [11, 12]. With the increasing number of examples of the complex interplay between bacterial physiology and virulence, we present here an investigation into the influence of PknG and GarA on virulence and metabolism of *M*. *tuberculosis* and *M*. *smegmatis* and identify stimuli for this signaling pathway.

*M*. *tuberculosis* and *M*. *smegmatis* are metabolically versatile and are able to synthesise all twenty proteinogenic amino acids, which may be critical to combat host strategies to starve intracellular bacteria of amino acids [16]. Although able to utilize inorganic nitrogen sources, *M*. *tuberculosis* shows a preference for amino acids such as glutamate, and these are also co-catabolised along with other carbon sources in axenic culture and in macrophages [17–22]. Genome analysis suggests that catabolism of glutamate is carried out by glutamate dehydrogenase (GDH) and metabolism via TCA cycle (alpha-ketoglutarate dehydrogenase, KDH), while glutamate synthase (GltS, also known as GOGAT) is the main route of glutamate biosynthesis [23] and this is supported by recent studies of *M*. *tuberculosis* and *M*. *bovis* BCG [24–26].

Co-catabolism of glutamate raises the challenge of maintaining the balance between carbon and nitrogen metabolism, particularly given host strategies to deprive intracellular bacteria of amino acids [16]. Glutamate is the major donor for transaminations so the intracellular glutamate pool must be preserved. A second challenge specific to the Actinobacteria is that the KDH complex (alpha-ketoglutarate dehydrogenase or oxoglutarate dehydrogenase complex) does not have a dedicated E2 subunit: the dihydrolipoyl transacetylase subunit is shared between the KDH and pyruvate dehydrogenase complexes [27], potentially requiring an additional degree of regulation of carbon metabolism. Bacteria commonly have multiple mechanisms to sense carbon and nitrogen status (starvation or sufficiency) including sensing key intracellular metabolites including oxoglutarate, glutamine, ATP, cyclic AMP, (p)ppGppp [28, 29]. The nitrogen sensor(s) of *M*. *tuberculosis* has yet to be identified [30], but compared to *Escherichia coli*, *M*. *tuberculosis* has only a single PII protein (nitrogen sensor) rather than two, and lacks the nitrogen-sensing two component system NtrB/C. Given the direct effects of GarA on the relevant enzyme activities (KDH, GDH and GltS), we reasoned that the system of PknG-GarA could fulfill this function in *Mycobacterium* spp. and other Actinobacteria and we set out to investigate the regulation of GarA by phosphorylation and its relationship to metabolism and virulence.

## Results

### GarA was required for establishment of infection of *M*. *tuberculosis* in macrophages and in mice

**Fig 1:**
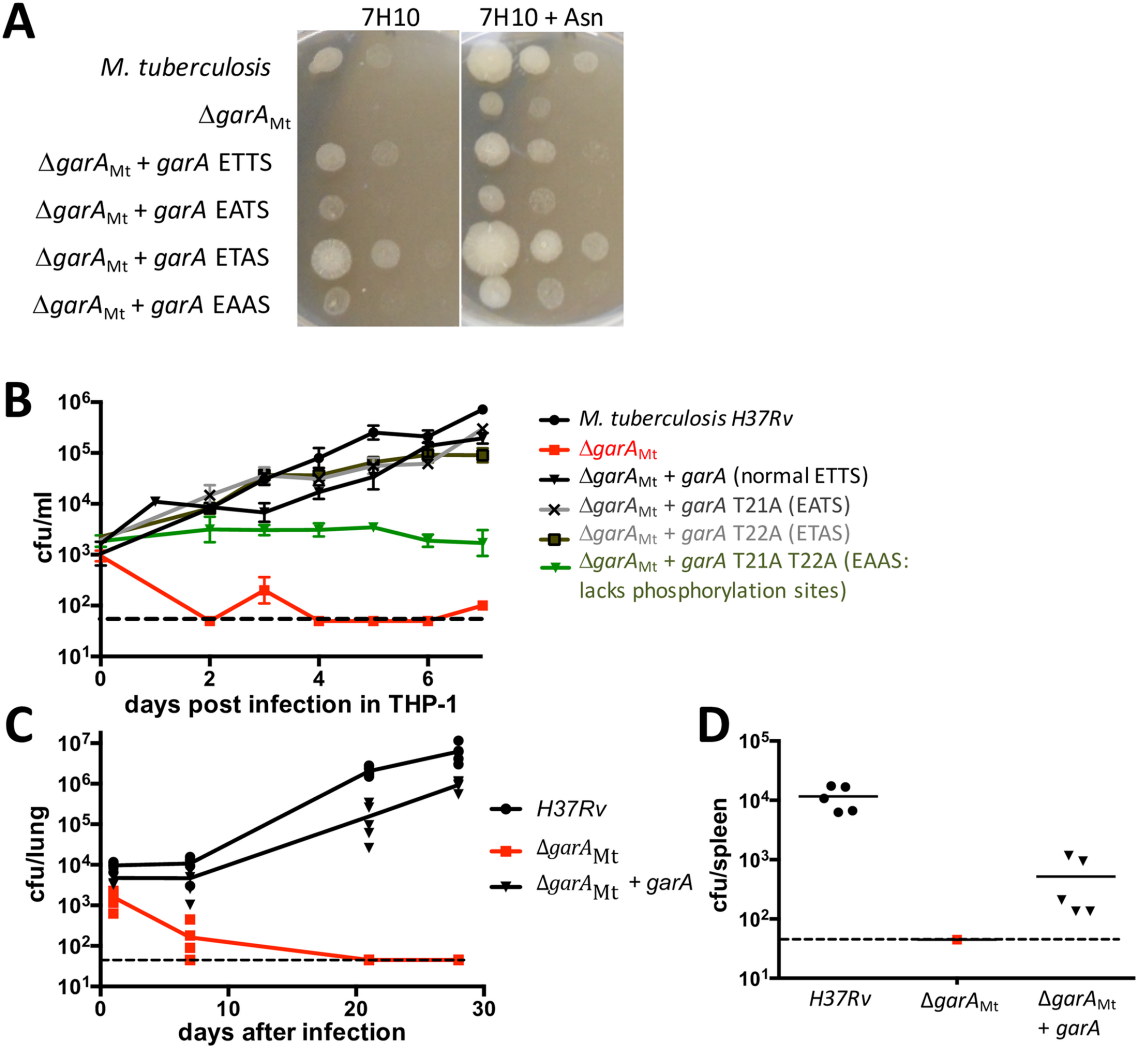
*garA* is required for growth of *M*. *tuberculosis in vitro*, survival in macrophages, and virulence in mice. (**A**) *M*. *tuberculosis* lacking *garA* was unable to grow on standard 7H10 medium unless supplemented with asparagine. Plasmid-borne *garA* restored the defect, but variants of *garA* with mutations at threonine 21 in the phosphorylation motif (ETTS) gave only partial complementation. Strains were grown in Middlebrook 7H9 plus 30 mM asparagine then washed and diluted in standard 7H9 and spotted onto standard 7H10 with or without 10 mM asparagine. Photographs are representative of at least 3 independent experiments. (**B**) *M*. *tuberculosis* lacking *garA* (red squares) had a defect in growth and survival in differentiated THP-1 cells compared to parental *M*. *tuberculosis H37Rv* (black circles). Re-introduction of GarA (black triangles) or variants of GarA lacking a single phosphorylation site (grey crosses and squares) restored growth but variant GarA lacking both phosphorylation sites (green triangles) did not. Data points show the mean and standard deviation from four replicates and are representative of two independent experiments. (**C**) *M*. *tuberculosis* lacking *garA* was avirulent in mice as it was eliminated from the lungs. BALB/C mice were infected intranasally with 10^5^ bacilli and bacterial burden was measured on days 1, 7, 21 and 28. Data points show the bacterial burden in individual animals. The bacterial burden of mice infected with Δ*garA*_Mt_ (red squares) was significantly lower than those infected with *M*. *tuberculosis H37Rv* (black circles), or complemented Δ*garA*_Mt_ (black triangles) at all time points from day 7 (p<0.005, t test). (**D**) *M*. *tuberculosis* lacking *garA* failed to disseminate to the spleen by day 28 (symbols match panel **C**). The minimum number of bacteria that could be detected was 45 CFU/organ, marked by a dashed black line in **C** and **D**.

We have previously constructed a conditional gene disruption mutant of *M*. *tuberculosis* and demonstrated that *garA* was essential in standard Middlebrook medium, which contains 3 mM glutamate, but dispensable when additional amino acid supplements were used (10 mM asparagine, glutamate or glutamine [9]). Using this knowledge, we have now constructed an unmarked *garA* deletion mutant Δ*garA*_Mt_ (Fig 1). This strain grew poorly on standard Middlebrook medium and growth was restored by addition of asparagine or reintroduction of *garA* (Figs 1A and S1). Asparagine was chosen rather than glutamate or glutamine because of good solubility and low influence on pH or buffering capacity.

Δ*garA*_Mt_ was tested for its ability to infect and replicate in THP-1 macrophages (ATCC TIB-202) and was unable to replicate (Fig 1B). Plasmid-encoded GarA complemented the defect (Fig 1B) but addition of 20 mM asparagine to the cell culture medium did not restore replication (S2 Fig), suggesting that intracellular *M*. *tuberculosis* could be in an environment with non-permissive asparagine concentration (below 10 mM). These results reinforce our earlier findings with the conditional mutant strain [9], which suggest that *garA* is essential for *M*. *tuberculosis* in macrophages and that this essentiality could be due to amino acid deprivation inside the phagosome.

Since Δ*garA*_Mt_ shows auxotrophy in axenic growth and attenuation in macrophages, we predicted it would be avirulent. To compare the virulence of Δ*garA*_Mt_ with parental *H37Rv*, equivalent inocula, as measured by colony forming units (CFU), were used for intranasal infection of immune competent BALB/c mice. Virulent *H37Rv* and the complemented strain replicated in the lungs and disseminated to the spleen within 28 days, whereas Δ*garA*_Mt_ failed to replicate within the lungs and no bacteria could be recovered from lungs or spleen after 21 days (Fig 1C & 1D). Notably, a clear confirmation of the attenuation was observed between the macrophage infection model and the mouse infection model, which is also compatible with the observed auxotrophy in axenic media. We conclude that *garA* was essential for virulence of *M*. *tuberculosis* in mice, which may be due to amino acid deprivation *in vivo* or because of the impact of primary metabolism on other aspects of virulence, such as stress tolerance [25]. According to our model, GarA and PknG act in the same pathway but with opposite effects on metabolism. Interestingly, loss of *garA* caused severe attenuation in our study (complete loss of replication and bacterial survival) whereas loss of *pknG* caused only partial attenuation in a previous study using two different mouse models [5].

### The phosphorylation sites on GarA were needed for growth of *M*. *tuberculosis*

We next turned our attention to the role of phosphorylation of GarA and the putative responsible kinase PknG. The normal role of phosphorylation is to “switch off” the activity of GarA by preventing it from binding to its enzyme targets [8]. The phosphorylation sites are found in a conserved ETTS motif in an unstructured N-terminal extension distinct from the forkhead-associated domain [31]. Variants of GarA lacking phosphorylation sites are functional for enzyme binding [31] but cannot be “switched off” by kinase activity, and thus might have an opposite effect on bacterial metabolism from *garA* knockout. We used variants of *garA* with mutations in the phosphorylation motif to complement the knockout strain Δ*garA*_Mt_ in order to examine the role of regulation of GarA by phosphorylation in *M*. *tuberculosis*. GarA variants lacking the first phosphorylation site, threonine 21 (EATS or EAAS at the motif) was less effective than normal GarA at restoring growth on Middlebrook medium (Fig 1A), despite confirmed expression (S3 Fig). In THP-1 macrophages GarA that lacks a single phosphorylation site (EATS or ETAS at the motif) restored growth of Δ*garA*_Mt_ in THP-1 macrophages but GarA lacking both phosphorylation sites (EAAS) failed to restore growth (Fig 1B). This suggests that the normal function of GarA requires regulation by phosphorylation. The single mutants EATS and ETAS led to slightly different levels of complementation in macrophages compared to axenic growth, which may reflect the different nutrient sources utilized in the two conditions.

As PknG is the only kinase reported to phosphorylate GarA at T21 (the first threonine in the GarA ETTS motif), disruption of *pknG* might be expected to alter phosphorylation of GarA, mimicking the effects of mutations to the GarA phosphorylation motif. Δ*pknG*_Mt_ has previously been reported to have a defect in survival in bone marrow derived mouse macrophages [11]. Unlike Δ*garA*_Mt_, Δ*pknG*_Mt_ was able to replicate in THP-1 macrophages, albeit 10-fold less than parental *M*. *tuberculosis* (S4 Fig). Taken together, these results support the importance of phosphorylation in regulating the function of GarA in *M*. *tuberculosis* both *in vitro* and during infection. Phosphorylation at both sites (T21 and T22) may be important, meaning that PknG and at least one other kinase may be involved, as is thought to be the case for the homologous system in *C*. *glutamicum* [32].

### Disruption of *garA* and *pknG* separately caused specific and opposing effects on nutrient requirements

Having established the importance of GarA for virulence, we probed the reasons for this essentiality and the extent of conservation between slow-growing *M*. *tuberculosis* and fast growing non-pathogenic model *M*. *smegmatis*. GarA binds to the same enzyme targets to promote the same effects on enzyme activity in both organisms [8, 31]. We predicted that *pknG* disruption (or disruption of GarA phosphorylation sites) would lead to an inability to catabolise glutamate (since excess unphosphorylated GarA would inhibit GDH and KDH), while *garA* disruption would lead to uncontrolled glutamate catabolism (since GltS would be less active and GDH and KDH would be uninhibited).

**Fig 2:**
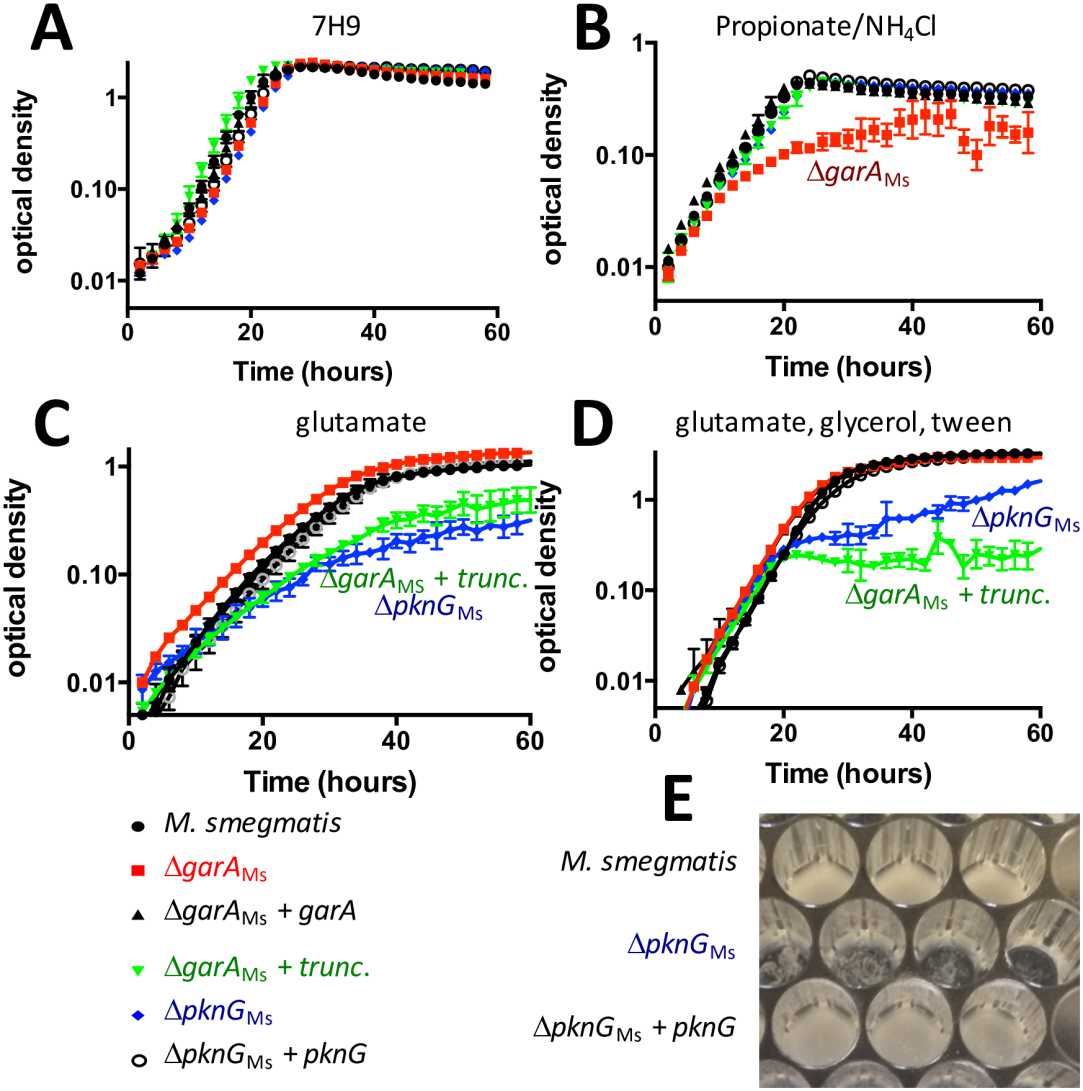
Disruption of *pknG* or removal of the phosphorylation motif of *garA* caused a nutrient-dependent growth defect in *M*. *smegmatis*. (**A**) All strains grew at the same rate on standard Middlebrook 7H9 medium. (**B**) Δ*garA*_Ms_ grew slower than wild type on minimal Sauton’s medium containing 20 mM propionate, 20 mM NH_4_Cl plus 0.05% tyloxapol, and this growth defect could be fully complemented by GarA lacking phosphorylation sites (truncated “trunc.” *garA*). (**C + D**) Δ*pknG*_Ms_ grew slower than wild type and formed clumps (inset photo) on medium containing glutamate as sole carbon (**C**) or nitrogen source (**D**) (minimal Sauton’s with either 30 mM glutamate plus tyloxapol, or 1% glycerol, 10 mM glutamate plus tyloxapol). Data plotted are the mean and standard deviation of at least 3 independent experiments. (**E**) Δ*pknG*_Ms_ formed clumps when glutamate was the sole carbon or nitrogen source. The photograph shows a microplate from growth curve (**D**) imaged at 60 hours. Growth of Δ*garA*_Ms_ complemented with phosphoablative GarA (EAAS) was equivalent to that of Δ*pknG*_Ms_ complemented with truncated GarA in all tested conditions so only the dataset for truncated GarA is shown for clarity.

We have previously studied the phenotype caused by *garA* deletion in *M*. *smegmatis* (Δ*garA*_Ms_). This strain grew normally on standard mixed medium (Fig 2A) but relied on external glutamate or related amino acids for growth and also showed differences from wild type in the ability to utilize a range of carbon sources [9]. Here we examined the ability of truncated GarA, which lacks the phosphorylation motif, to complement the growth defect. Truncated GarA and EAAS GarA fully complemented the growth defect of Δ*garA*_Ms_ on media lacking glutamate, indicating that these variant proteins are functional in stimulating glutamate production and preventing glutamate catabolism (Fig 2B). However, when glutamate was the only source of carbon (Fig 2C) or nitrogen (Fig 2D), strains lacking *pknG* or strains expressing non-phosphorylatable GarA formed clumps (Fig 2E) and grew poorly compared to the parent strain. The nutrient-specific growth phenotypes recorded in microplates (Fig 2) were also apparent when the same strains were cultured in flasks (S5 Fig).

**Fig 3:**
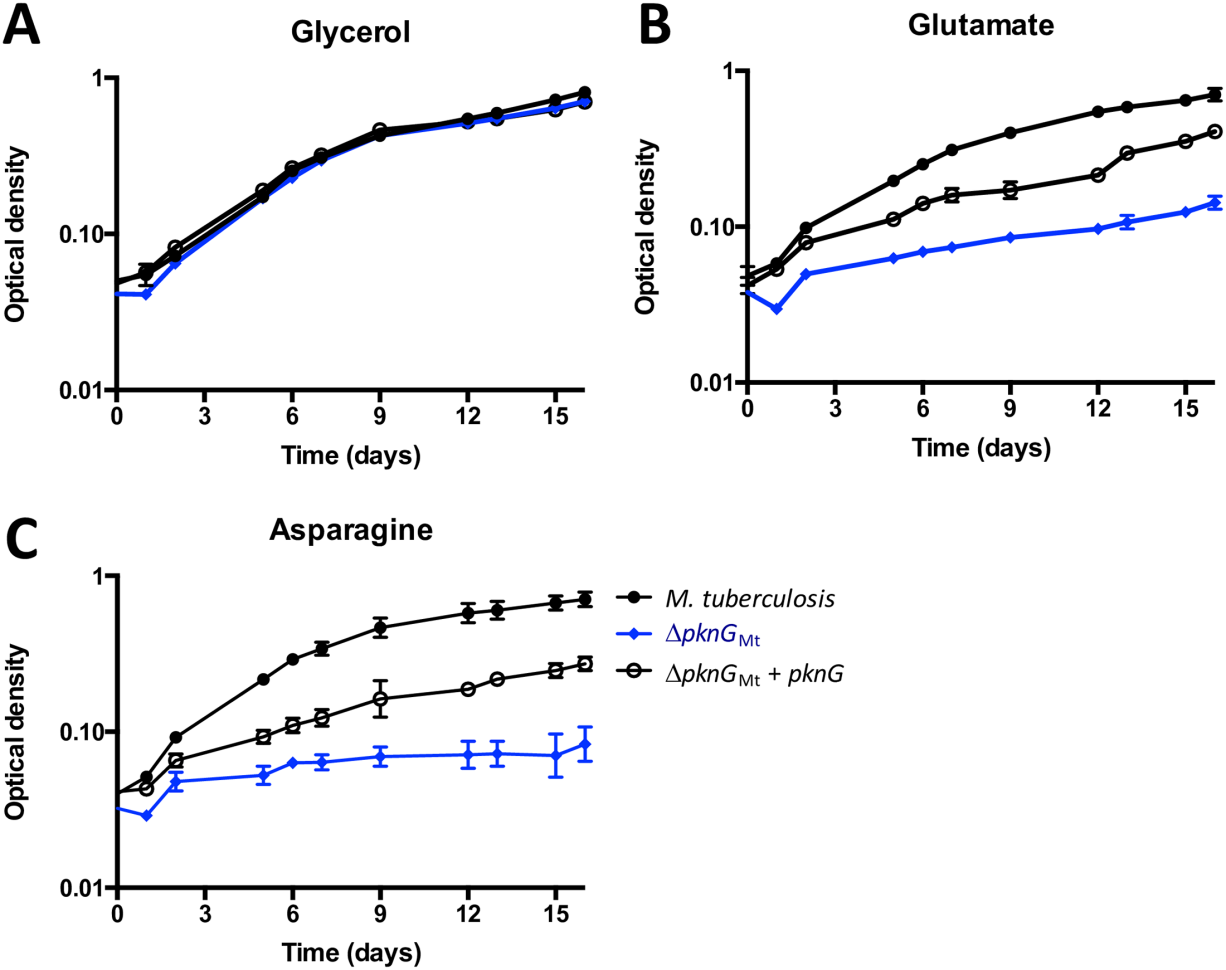
*pknG* disruption in *M*. *tuberculosis* caused a nutrient-dependent growth defect. (**A**) All strains grew at the same rate on minimal medium supplemented with glycerol 0.2%. Δ*pknG*_Mt_ (blue diamonds) grew more slowly than wild type *M*. *tuberculosis* (black circles) when the sole carbon source was (**B**) glutamate 10 mM or (**C**) asparagine 10 mM. Plasmid-encoded PknG (empty circles) partially restored the growth defect. Graphs show the mean and standard deviation of three independent experiments.

Similarly, Δ*pknG*_Mt_ showed no growth defect compared to parental *M*. *tuberculosis* on minimum medium supplemented with glycerol (Fig 3A) or glucose or acetate (S6 Fig), but had a specific growth defect when asparagine or glutamate were used as the sole carbon source in liquid culture (Fig 3B & 3C). Reintroduction of *pknG* restored PknG expression (S7 Fig) and improved growth on asparagine and glutamate (Fig 3B & 3C), although full restoration of growth was not achieved, possibly due to deleterious effects of PknG over-expression.

The nutrient-specific phenotypes of *M*. *tuberculosis* and *M*. *smegmatis* gene knockouts were both specific to amino acid metabolism: *garA*-disrupted strains required glutamate or asparagine for growth, while *pknG*-disrupted strains had a defect in utilization of glutamate or asparagine. To investigate the likely conservation of function of the regulatory pathway between *M*. *tuberculosis* and *M*. *smegmatis* we used *M*. *tuberculosis pknG* and *garA* to complement the growth defects of *M*. *smegmatis* mutants (S8 Fig). Our results suggest conservation of function of PknG and GarA between a fast-growing saprophyte and a slow-growing pathogen (other differences between these organisms have been reviewed [33–35]).

In summary, the nutrient-specific phenotypes of *pknG*- and *garA*-disrupted *M*. *smegmatis* and *M*. *tuberculosis* support a role for these proteins in regulating amino acid metabolism.

### PknG was needed for GarA phosphorylation in *M*. *smegmatis* and *M*. *tuberculosis*

**Fig 4:**
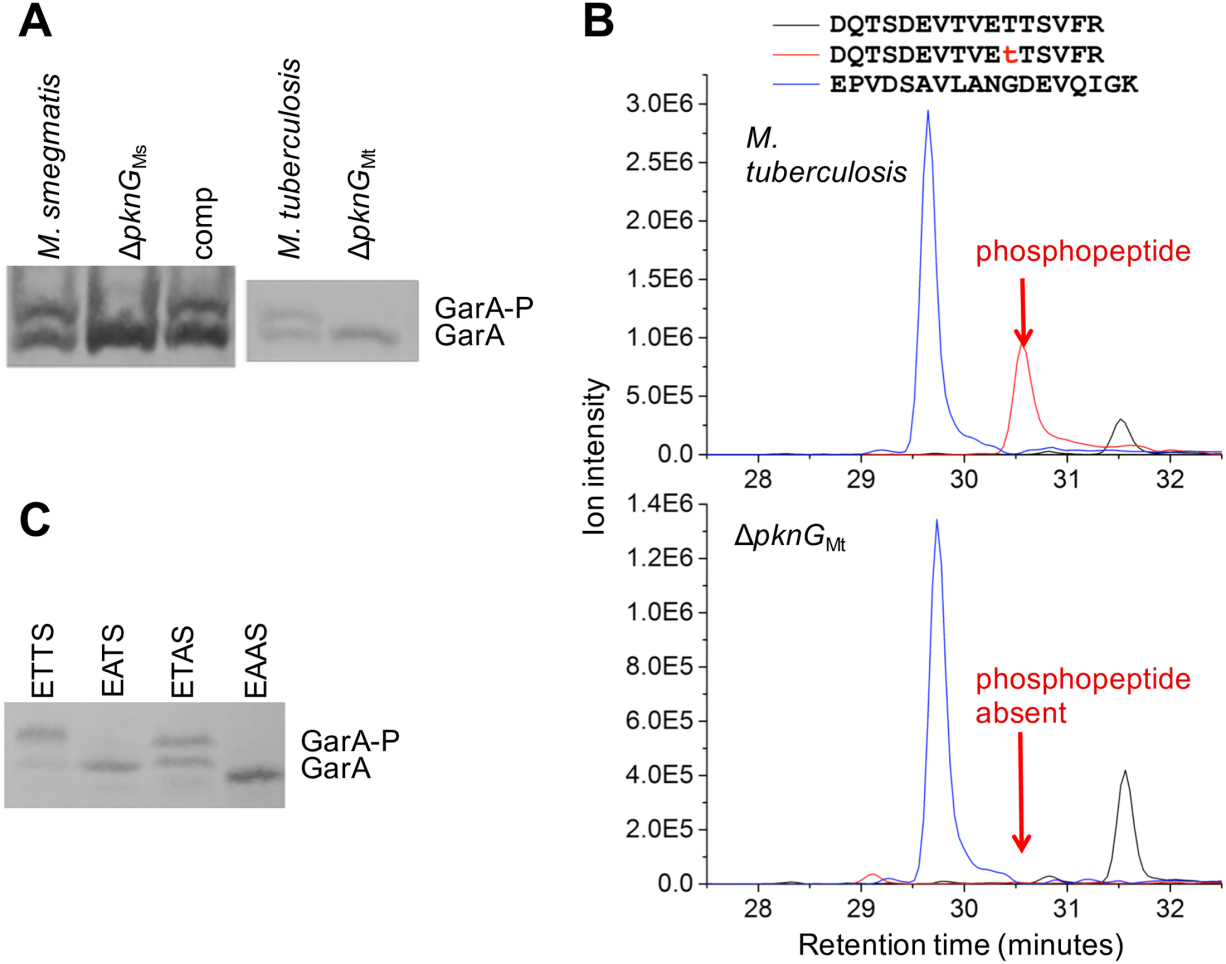
Investigation of GarA phosphorylation in *M*. *smegmatis* and *M*. *tuberculosis*. (**A**) Addition of Phos-tag reagent to SDS-PAGE allowed separation of phosphorylated GarA (GarA-P) from GarA. Cell extracts were prepared from *M*. *smegmatis* and *M*. *tuberculosis* wild type and *pknG* deletion strains and complemented strains. (**B**) The same cell extracts of *M*. *tuberculosis* wild type and *pknG* deletion strain were analysed by LC-MS/MS to detect the tryptic peptide of GarA carrying the ETTS phosphorylation motif. The abundance of the peptide with no phosphorylation is shown in black and the T21-phosphorylated peptide is shown in red. A peptide from another part of the protein was also measured as a control (blue). (**C**) A reporter strain of Δ*garA*_Ms_ carrying plasmids encoding hexahistidine-tagged *garA*, or variants of *garA*, confirmed that most phosphorylation occurred at the first threonine in the ETTS motif. GarA from cell lysates was visualised by Western blotting. Images shown are representative of three or more independent replicates.

To investigate kinase(s) that phosphorylate GarA in live mycobacteria and the stimuli that lead to kinase activity, we developed methods to distinguish phosphorylated GarA from the unphosphorylated form in cell extracts. Hexahistidine-tagged GarA shows a shift in mobility in SDS PAGE upon phosphorylation [8] but for untagged GarA this shift was too minor for reliable separation (for example S3 Fig shows a single band though Fig 4B demonstrates a mixture of phosphorylated and unphosphorylated GarA). Here we used three methods to determine whether GarA is phosphorylated in cells: (i) use of the Phos-tag reagent to retard the mobility of phosphorylated GarA in SDS PAGE of cell extracts from *M*. *tuberculosis* and *M*. *smegmatis* (Fig 4A), (ii) development of LC-MS/MS protocols to detect GarA in cell extracts of *M*. *tuberculosis* (Fig 4B), (iii) replacement of endogenous GarA with a hexahistidine-tagged version in *M*. *smegmatis* (Fig 4C).

*M*. *smegmatis* and *M*. *tuberculosis* growing in standard media contained two forms of GarA suggesting that cells contained a mixture of phosphorylated and unphosphorylated GarA (Fig 4A). In *M*. *smegmatis* lacking *pknG* the upper band was missing but could be restored by the introduction of plasmid-borne GarA, showing that PknG was the main kinase responsible for GarA phosphorylation. In *M*. *tuberculosis* lacking *pknG* the upper band was also missing, suggesting that PknG could also be responsible for phosphorylating *M*. *tuberculosis* GarA. Reintroduction of *pknG* to Δ*pknG*_*Mt*_ did not restore GarA phosphorylation, despite strong overexpression of *pknG* (S7 Fig). Non-physiological expression levels may influence GarA phosphorylation (see below for investigation into the conditions and stimuli that provoke phosphorylation).

To seek clarification about whether PknG may phosphorylate GarA in *M*. *tuberculosis* we decided to investigate the specific site of GarA phosphorylation. Several kinases have been reported to phosphorylate purified GarA at the second threonine (T22) while PknG is the only kinase shown to phosphorylate the first threonine (T21) [8, 36]. LC-MS/MS can distinguish between phosphorylation at T21 or T22 (S9 & S10 Figs). We have previously enriched phosphorylated GarA from cell extracts for detection by LC-MS/MS [8]. Here we developed a protocol avoiding enrichment to detect the various forms of GarA in cell extracts of *M*. *tuberculosis*. We used this protocol to determine the relative abundance of the three forms of GarA by comparison with peptide standards (unphosphorylated, T21-phosphorylated, and T22-phosphorylated). In wild type cells all three forms were detected (Figs 4B and S10) but the concentration of T22-phosphorylated form was always too low to quantitate reliably. However, in cell extracts of the *M*. *tuberculosis pknG* mutant strain there was no detectable T21-phosphorylated GarA, supporting the suggestion from Fig 4A that PknG may phosphorylate GarA in *M*. *tuberculosis*.

The equivalent peptides of *M*. *smegmatis* GarA were less amenable to mass spectrometry and so we created a reporter strain: Δ*garA*_Ms_ + *His*_*6*_*garA*, in which the *garA* deletion strain Δ*garA*_Ms_ [9] is complemented by hexahistidine-tagged GarA (S11 Fig). We also generated variants with mutations in the phosphorylation motif of GarA ETTS. Western blotting showed that only those GarA variants lacking the phosphorylation site for PknG were predominantly unphosphorylated (Fig 4C). In summary, the data from Fig 4A–4C clearly demonstrate that PknG is the main kinase responsible for phosphorylation of GarA in *M*. *smegmatis* whereas phosphorylation by other kinase(s) may occur at lower levels, similar to the findings in *C*. *glutamicum* [32]. We also provide three independent lines of evidence to show that PknG may phosphorylate GarA in *M*. *tuberculosis*: site specificity (T21 phosphorylation in cells), loss of phosphorylation in Δ*pknG*_Mt_, and conservation of function since *M*. *tuberculosis pknG* was able to complement the growth defect of *M*. *smegmatis pknG* knockout (S8 Fig).

### Amino acids and optimal carbon sources triggered rapid phosphorylation of GarA

We next sought to identify the specific environmental signals that trigger phosphorylation or dephosphorylation of GarA. Since the active form of GarA is the unphosphorylated form, we predicted that this form would predominate in conditions where *garA* is essential, such as during amino acid deprivation. We used mass spectrometry to investigate GarA phosphorylation in *M*. *tuberculosis* and found only the unphosphorylated form in PBS-starved *M*. *tuberculosis* compared to a mixture in cells grown on standard media (S12 Fig). This trend of GarA phosphorylation in optimal medium but a lack of phosphorylation upon amino-acid starvation is similar to observations made on the homologous protein in *C*. *glutamicum* [37].

**Fig 5:**
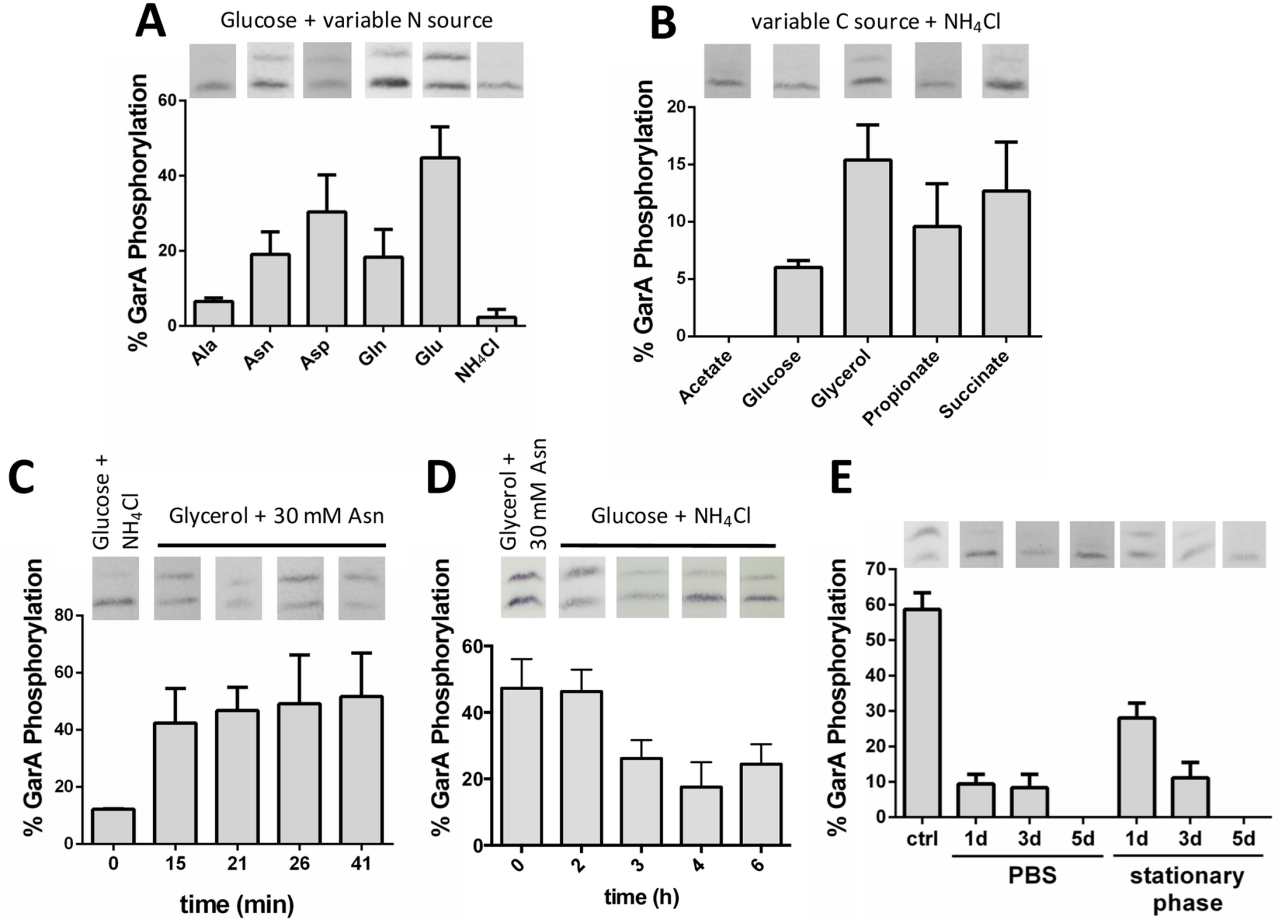
GarA phosphorylation in *M*. *smegmatis* was regulated by nutrient availability. The reporter strain of *M*. *smegmatis* was cultured in different media and cell lysates analysed by Western blot and densitometry. (**A**) Glutamate and related amino acids triggered phosphorylation of GarA: the nitrogen source is indicated and the carbon source was glucose. (**B**) The supplied carbon source affected phosphorylation of GarA: the carbon source is indicated and the nitrogen source was NH_4_Cl. (**C**) Phosphorylation of GarA occurred rapidly when cells were cultured in poor medium and then given supplementary nutrients (initially 1% glucose with 10 mM NH_4_Cl and 0.05% tyloxapol, then 1% v/v glycerol and 30 mM asparagine were added at time zero). (**D**) Dephosphorylation of GarA occurred slowly when cells switched from rich to poor medium (initially 1% glycerol with 30 mM asparagine and 0.05% Tween 80 then switched to 1% glucose with 10 mM NH_4_Cl and 0.05% tyloxapol). (**E**) GarA was predominantly unphosphorylated when *M*. *smegmatis* were in stationary phase or starved in PBS. The reporter strain of *M*. *smegmatis* was grown in Sauton’s medium with shaking for 5 days. For the starvation experiment exponentially growing *M*. *smegmatis* were washed with PBS and incubated in PBS with 0.05% tyloxapol for 5 days. Values represent mean and standard deviation of at least three independent replicates.

We then used the reporter strain of *M*. *smegmatis* to analyse a range of carbon and nitrogen sources separately for their effects on GarA phosphorylation (Fig 5A and 5B). Strikingly, the nutrients that led to the most phosphorylation (Fig 5A), are the amino acids that rescue the growth defect of Δ*garA*_Mt_ and Δ*garA*_Ms_: glutamate, aspartate, glutamine and asparagine.

When carbon sources were compared there was least phosphorylation during growth on acetate or glucose (Fig 5B). The correlation between the extent of GarA phosphorylation and the severity of growth phenotype of Δ*garA*_Ms_ was weaker when comparing carbon sources, suggesting that there may be other sensory/regulatory input(s) that remain to be identified. Thus we conclude that nutrients are likely stimuli for PknG activity, and, at least in *M*. *smegmatis*, glutamate and related amino acids are the most important. Notably, the sensory mechanism remains to be identified and there are also likely to be additional stimuli influencing kinase activity.

In principle, reversible phosphorylation of GarA could allow cells to respond rapidly to changing nutrient availability. Since GarA interacts directly with enzymes of central carbon and nitrogen metabolism this would allow a more rapid response than alterations in gene expression level. To investigate the dynamics of adaption we grew the reporter strain on media promoting low or high phosphorylation of GarA and then exchanged the medium at mid-log phase, monitoring GarA phosphorylation until it had stabilized. Addition of glycerol/asparagine to a culture grown in medium containing glucose/ammonium chloride led to GarA phosphorylation within the shortest time period that could be sampled with accuracy (15 minutes) (Fig 5C). By contrast, when cells were transferred from standard Sauton’s medium to minimal Sauton’s medium, reductions in GarA phosphorylation were only seen after three hours, suggesting that dephosphorylation occurred slowly if at all (Figs 5D and S13). Similarly to *M*. *tuberculosis*, we observed only unphosphorylated GarA in starved or stationary phase *M*. *smegmatis* (Fig 5E). Our observations suggest that the PknG—GarA system may allow cells to adapt rapidly to an increase in amino acid availability: glutamate or related amino acids would stimulate PknG to phosphorylate GarA and hence enable glutamate catabolism. However, adaptation to nitrogen starvation may take longer and could involve new protein synthesis or protein dilution through cell division.

### GarA was required for preservation of intracellular glutamate during extended stationary phase

**Fig 6:**
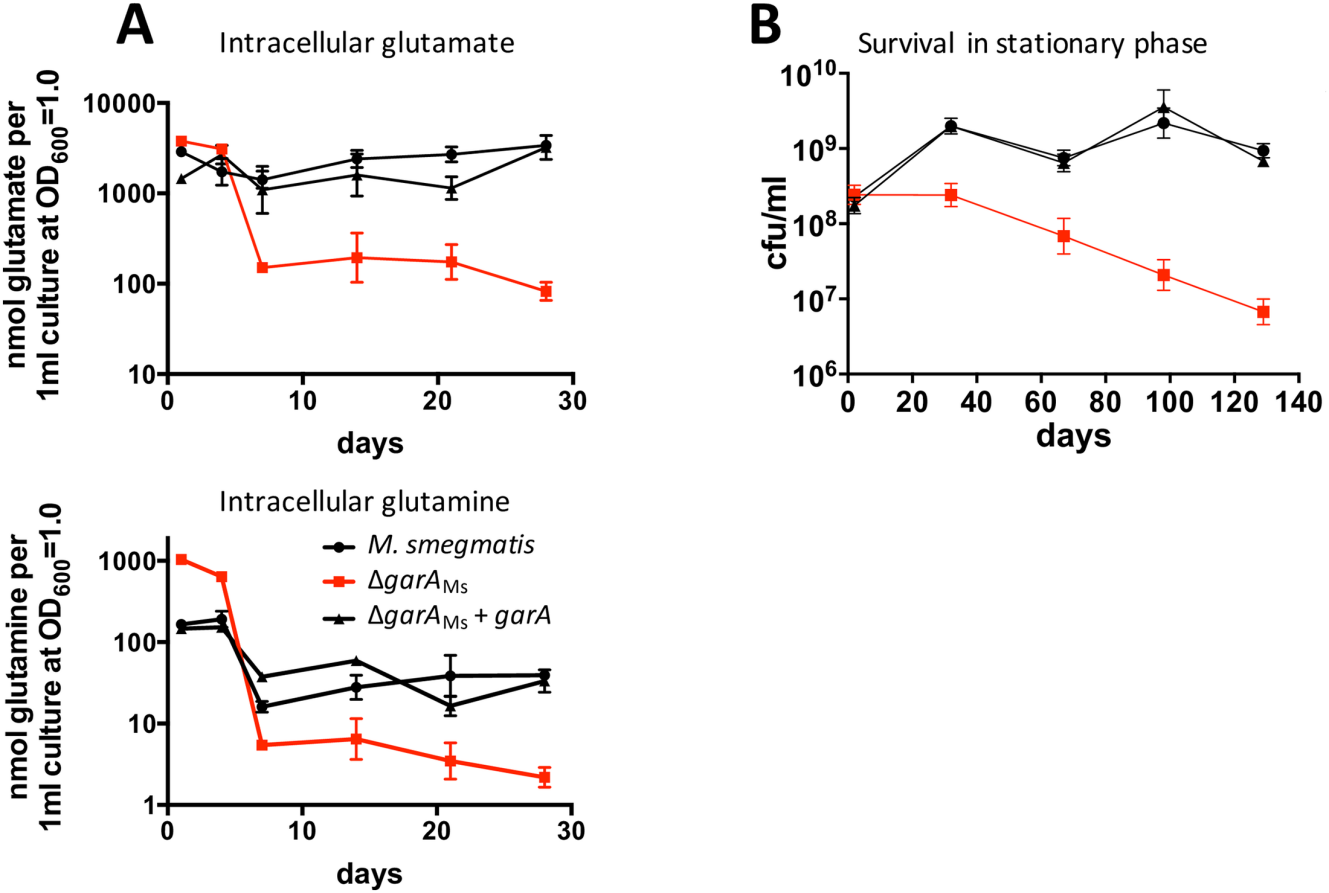
GarA was required during stationary phase for the maintenance of intracellular glutamate and for survival. (**A**) *M*. *smegmatis* lacking *garA* failed to maintain intracellular glutamate and glutamine pools during extended stationary phase in 7H9 medium. Intracellular glutamate and glutamine were measured for wild type *M*. *smegmatis* (black circles), Δ*garA*_Ms_ (red squares), and complemented Δ*garA*_Ms_ (black triangles). Inset graphs show intracellular metabolites for the same experiment at day 28 (**B**) *M*. *smegmatis* lacking *garA* gradually lost viability during prolonged stationary phase. Cells were cultured in 7H9 medium over a time period of five months. Aliquots were withdrawn at regular intervals and surviving cells were plated on 7H10 to calculate CFU ml^-1^. All experiments were repeated at least 3 times and data show the mean with standard deviation.

Having established that GarA is predominantly in the active, unphosphorylated form during starvation and stationary phase in *M*. *tuberculosis* and *M*. *smegmatis* (S12 Fig and Fig 5E), we used a metabolomics approach with *M*. *smegmatis* to test the specific effects of *garA* knockout on intracellular metabolites. Since GarA stimulates glutamate synthase activity and inhibits enzymes involved in glutamate catabolism, we predicted that Δ*garA*_Ms_ might have a lower concentration of intracellular glutamate compared to wild type *M*. *smegmatis*. We used a targeted mass spectrometry approach to monitor the intracellular concentration of glutamate and 39 additional metabolites of central carbon metabolism in stationary phase cultures. Of the 40 metabolites analysed, glutamate and glutamine showed the greatest difference between Δ*garA*_Ms_ compared to wild type. In wild type cells the concentration of glutamate was maintained at a relatively steady level throughout 28 days while the concentrations of glutamine and many other metabolites declined during the first 7 days and were then steady (Figs 6A & S14). As predicted, Δ*garA*_Ms_ had lower intracellular glutamate in stationary phase (from day 7 onwards). The intracellular concentration of glutamine was transiently elevated during entry of Δ*garA*_Ms_ into stationary phase and then declined from day 7 onwards (Fig 6A). The decline in glutamate and glutamine in extended phase could be due to catabolism through uninhibited GDH and KDH. After 28 days the Δ*garA*_Ms_ strain began to show loss of viability. Metabolite sampling was discontinued and viability was monitored for 10 further weeks, by which point cultures of Δ*garA*_Ms_ contained 100-fold fewer CFU ml^-1^ than wild type *M*. *smegmatis* (Fig 6B). The depletion of intracellular glutamate in stationary phase Δ*garA*_Ms_ and the defect in long-term survival provides a functional demonstration of our predicted model of metabolic regulation and highlights the importance of glutamate homeostasis for bacterial viability.

### Metabolome analysis revealed amino acid metabolism as the main target of regulation by GarA and PknG in *M*. *smegmatis*

**Fig 7:**
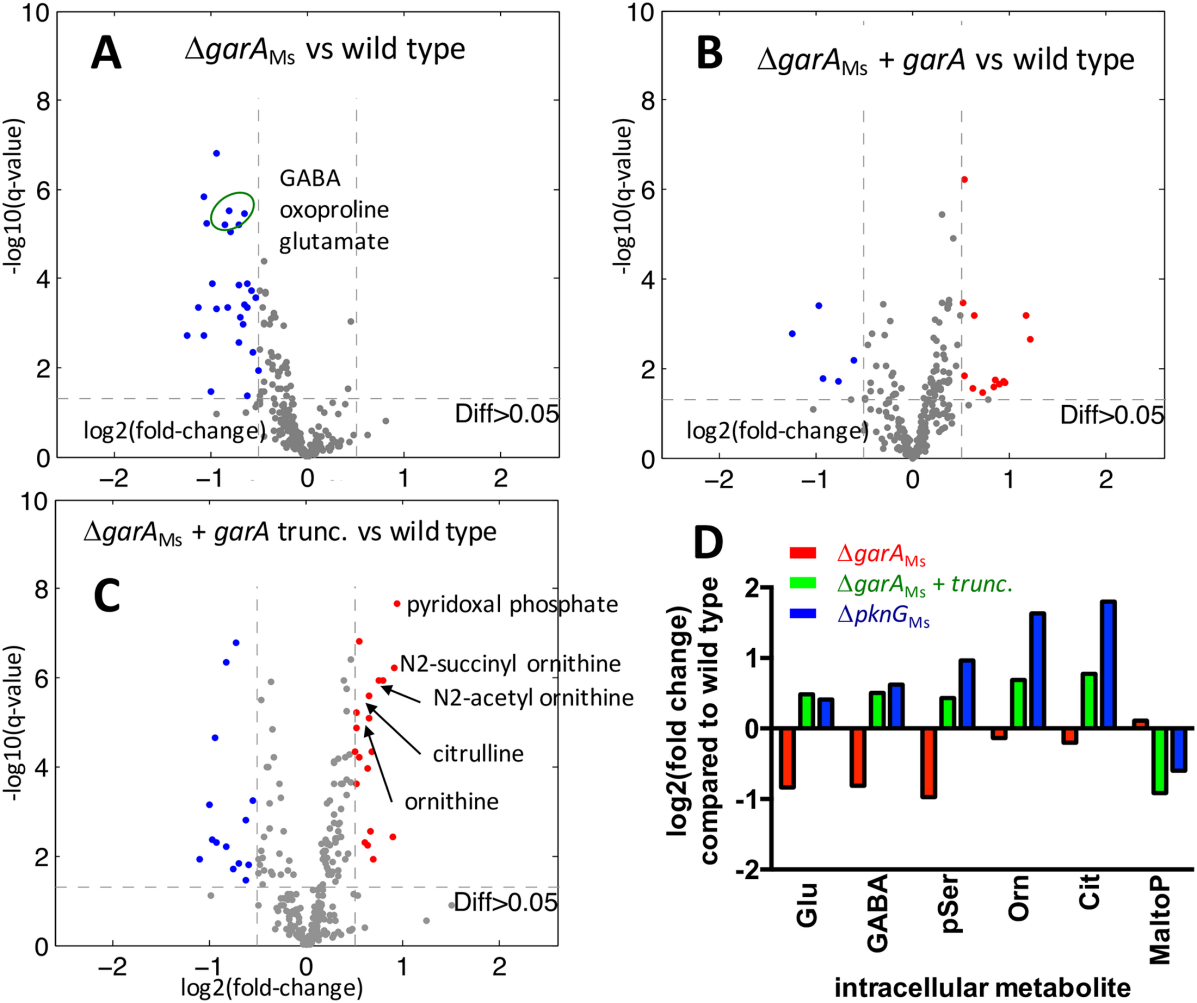
Deletion of *garA* or disruption of GarA phosphorylation caused changes in intracellular glutamate and other changes in the metabolome of *M*. *smegmatis*. Intracellular metabolites from wild type *M*. *smegmatis* and mutants were analysed by mass spectrometry using an untargeted metabolomics approach. *M*. *smegmatis* lacking *garA* has lower intracellular glutamate and metabolites related to glutamate than the parental strain (**A**) but plasmid encoded GarA reversed this change (**B**). *M*. *smegmatis* expressing truncated GarA that lacks phosphorylation sites had higher intracellular ornithine than wild type (**C**). Intracellular glutamate concentrations for the mutant strains are compared to wild type in panel (**D**), together with those metabolites that were significantly changed in >1 mutant strain but not in complemented strains. pSer is O-phosphoserine, Orn is ornithine, Cit is citrulline, MaltoP is maltopentaose. (**A-C**) Each point on the scatter plots represents a single metabolite. Metabolites with the greatest fold-change and statistical significance are highlighted (log2(fold change)>0.5 and q-value<0.05), thresholds marked as dashed lines on graphs): metabolites at lower concentration are blue and those at higher concentration are red. These data were taken from cells growing in Middlebrook 7H9 in early exponential phase and represent the mean from at least 3 independent experiments.

Disruption of *pknG* in *M*. *tuberculosis* has previously been shown to perturb intracellular glutamate and glutamine levels [5]. To examine the effect of *garA* disruption or *pknG* disruption on wider cell metabolism we grew *M*. *smegmatis* and variant strains for untargeted metabolome analysis of about four hundred annotated metabolites by mass spectrometry. An unbiased comparison of the metabolomes of Δ*garA*_Ms_ with the parent and complemented strains identified a set of 15 metabolites with lower concentration in Δ*garA*_Ms_ (log2(fold change)>0.5, and q-value<0.05) (Fig 7A and Table 1). Eight of the fifteen significantly changed metabolites were amino acids or intermediates in amino acid biosynthesis. Striking reductions were seen in the intracellular concentrations of glutamate and two direct products of glutamate: GABA and oxoproline/pyroglutamate (Fig 7A), and these changes were reversed by plasmid-borne *garA* (Fig 7B). Extracellular metabolites were also analysed but significant differences were not found (S15 Fig).

**Table 1:** Summary comparing the intracellular metabolome of mutant strains with parental *M*. *smegmatis*. Metabolites that are significantly changed in more than one strain are in **bold** type. Metabolites related to amino acid metabolism are highlighted with asterisks *. Metabolites are listed in order of fold-change compared to *M*. *smegmatis*, and those that were >2-fold changed are shaded.

	Δ*garA*_Ms_	Δ*garA*_Ms_ + *garA* EAAS	Δ*pknG*_Ms_
**Higher concentration than *M*. *smegmatis***	None	*O-Acetyl-L-homoserine	*Ornithine
		Lactose/Maltose/Trehalose	*Citrulline
		Pyridoxal 5'-phosphate	GMP
		*N2-Acetyl-L-ornithine	*O-Phosphoserine
		*Citrulline	GDP
		*Ornithine	*Betaine aldehyde
		Phosphatidylethanolamine	*(R)-2,3-Dihydroxy-3-methylpentanoate/(R)-Pantoate
		*Proline	methyl octadecanoate;10-methylstearic acid
		FAD	IMP
		Hydrogenobyrinate a,c diamide	di-methyl behenic acid;tetracosanoate (n-C24:0)
		Ribose phosphate isomers	*4-Aminobutanoate
		*Phenylacetaldehyde	dimethyl lignoceric acid;hexacosanoate (n-C26:0)
			*3-Phosphohydroxypyruvate
**Lower concentration than in *M*. *smegmatis***	1D-myo-inositol 2-Acetamido-2-deoxy-D-glucopyranoside	*Thiocyanate	2-C-methyl-D-erythritol 4-phosphate
	(E,E,E,E,E,E,E,Z,Z) decaprenyl phosphate	**Maltopentaose**	Glycerol
	*LL-2,6-Diaminoheptanedioate;meso-2,6-Diaminoheptanedioate	*Glucosamine phosphate (isomers)	Citrate;Isocitrate
	*O-Phosphoserine	Riboflavin	*N-Carbamoyl-L-aspartate
	sn-Glycero-3-phosphoethanolamine	4-Amino-5-hydroxmethl-2-methylpyrimidine	*N2-Succinyl-L-glutamate
	*Glutamate (or isomers)	*2,3-dihydroxy-3-methylpentanoate or pantoate	*Aspartate
	*4-Aminobutanoate	*2-isopropylmaleate	Coenzyme A
	N2-Formyl-N1-(5-phospho-D-ribosyl)glycinamide		gamma-L-Glutamyl-L-cysteine
	N-Pantothenoylcysteine		Dihydroorotate
	*N-Succinyl-2-L-amino-6-oxoheptanedioate		*Alanine
	*Aspartate		**Maltopentaose**
	*5-Oxoproline		*L-Aspartate 4-semialdehyde
	Phosphatidylglycerophosphate (dituberculostearoyl, C19:0)		
	*Serine		
	D-Alanyl-D-alanine		

To examine the impact of disrupting GarA phosphorylation, we next analysed the intracellular metabolites of Δ*garA*_Ms_ carrying non-phosphorylatable GarA and Δ*pknG*_Ms_ (Tables 1 and S1, Figs 7C and S16). We predicted elevation in intracellular glutamate when GarA cannot be phosphorylated, a reversal of the glutamate deficiency when *garA* is deleted. Intracellular glutamate was indeed significantly higher (1.3-fold change, q-value<0.0001, Fig 7D, S1 Table) but below our chosen threshold for inclusion in Table 1 (log2(fold change)>0.5). The majority of the metabolites that were significantly changed were amino acids or involved in amino acid metabolism, notably intermediates of arginine biosynthesis (ornithine, citrulline), which were elevated in mutant strains. The wide reaching changes in amino acid metabolism could be consequences of perturbed glutamate metabolism, the central hub of NH_3_ transfer. Beyond amino acid metabolism, we cannot differentiate whether changes in metabolite concentrations are indirect consequences of altered physiology and altered amino acid metabolism or indicative of other GarA/PknG targets.

Previously *pknG* deletion has been shown to cause raised intracellular glutamate and glutamine in *M*. *tuberculosis* [5] and raise glutamate production by *C*. *glutamicum* [37], whereas *garA* deletion abolished glutamate production by *C*. *glutamicum*. Our metabolome analysis of *M*. *smegmatis* reinforces the functional link between PknG and GarA and their role in regulating amino acid metabolism in Actinobacteria, which is further supported by perturbations in amino acid metabolism seen in the metabolome of *pknG*-disrupted *M*. *bovis* BCG (S7 Table). We chose a medium in which all strains grew at the same rate and in which the wild type strains of *M*. *smegmatis and M*. *bovis BCG* normally contain a mixture of phosphorylated and unphosphorylated GarA. We observed that perturbation of *pknG* caused changes in the concentrations of intracellular metabolites despite the absence of an obvious growth defect. Together with Figs 1–4, this highlights the relevance of phosphorylation for regulation of GarA function, and the importance of proper regulation of phosphorylation.

## Discussion

This work has validated the biological significance of the PknG—GarA signaling pathway that is needed for virulence of pathogenic *M*. *tuberculosis* and for balanced nutrient utilization of both *M*. *tuberculosis* and non-pathogenic *M*. *smegmatis*. GarA exhibits a key characteristic of a signaling protein in a kinase pathway, namely variable levels of phosphorylation as cells respond to different environments. Genetic disruptions that led to a loss of responsiveness (either permanent activation or permanent inactivation) caused metabolic changes and loss of virulence. This requirement for responsiveness may highlight the changing environmental conditions that are encountered by the pathogen during infection and also the importance of proper regulation of this central node of metabolism and how vulnerable it is to any kind of disruption. This adds to the growing body of evidence linking nutritional adaptation to virulence of *M*. *tuberculosis* and other pathogens.

The role of PknG in metabolic control via GarA has previously been supported mainly by experiments on purified proteins but here it was demonstrated in cells. It is interesting to note that although PknG appeared to be the main kinase responsible for phosphorylating GarA, we found indications that other kinase(s) could be involved in both *M*. *tuberculosis* and *M*. *smegmatis*, similar to the observation that multiple kinases are involved in *C*. *glutamicum* [32].

**Fig 8:**
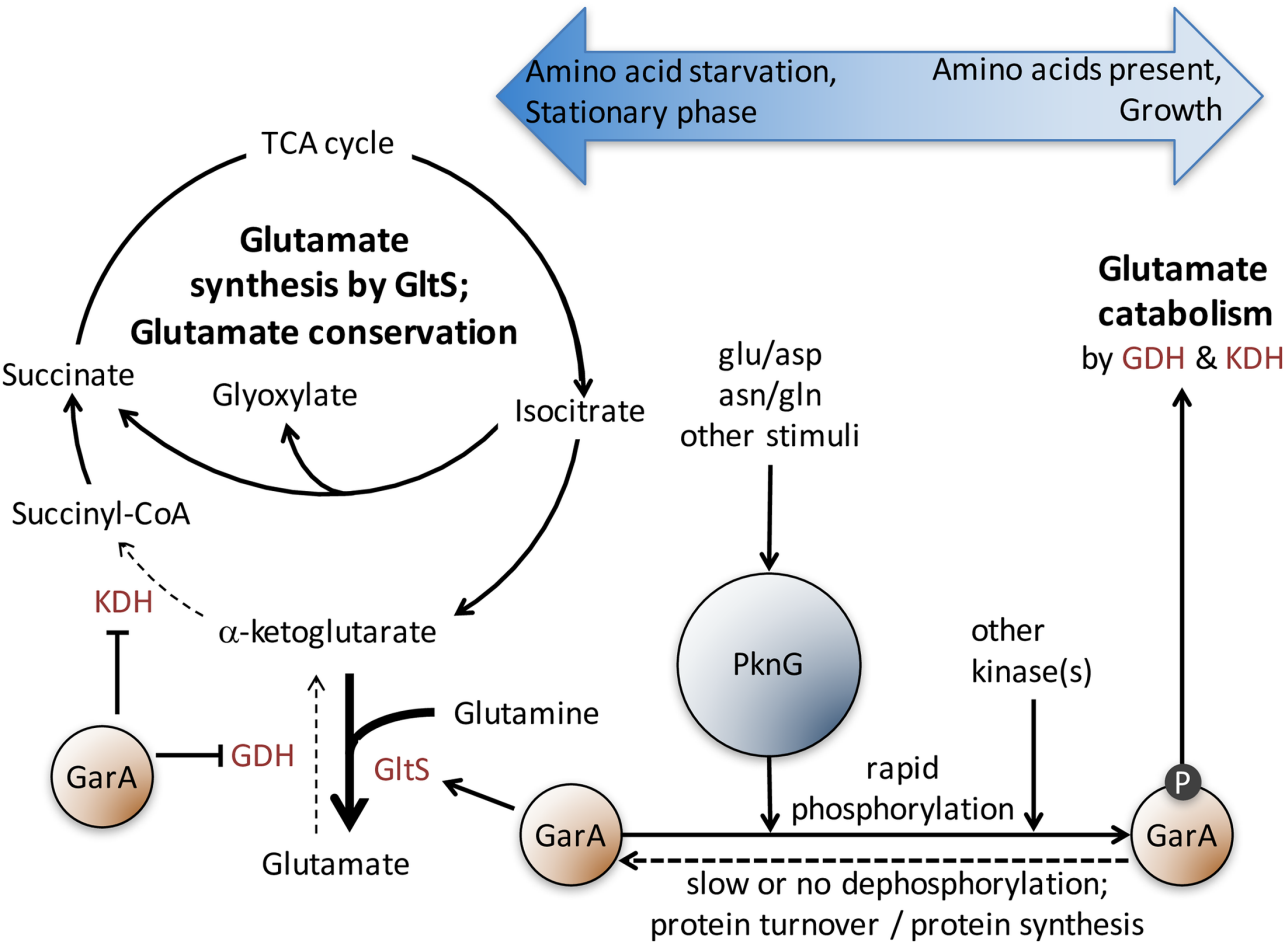
A model depicting control of the TCA cycle and glutamate metabolism by PknG and GarA. When PknG activity is low, unphosphorylated GarA activates glutamate synthesis and inhibits glutamate catabolism by direct binding to the relevant enzymes. When PknG activity is stimulated, for example by glutamate and aspartate, GarA become phosphorylated, causing a shift in metabolism towards glutamate catabolism. KDH is the alpha-ketoglutarate dehydrogenase complex, GDH is glutamate dehydrogenase, GltS is glutamate synthase.

This level of experimental validation of kinase function has rarely been performed for other bacterial serine/threonine protein kinases. The prevailing view of serine/threonine phosphorylation as a transient, reversible signaling event that occurs upon cellular stimulation derives largely from comparison with eukaryotic kinases, whereas little is known of the stimuli or kinetics of kinase activation in bacteria. Not only are the stimuli of bacterial kinases largely unidentified, but the macronutrients sensed by *M*. *tuberculosis* are also unknown [30]. The identification of externally supplied glutamate, aspartate, asparagine and glutamine as stimuli of PknG activity (Fig 5) begins to address these important questions, although the molecular mechanisms remain to be elucidated. Our data also indicate that there are other additional sensory input(s) both in the form of other kinases acting on GarA and other stimuli activating PknG (Fig 8). Given the large multi-domain structure of PknG it is a plausible candidate for integrating multiple sensory inputs to regulate metabolism precisely. Amino acids had not previously been proposed as activators of PknG, but alternative proposals include cellular redox status, since mutation of the rubredoxin domain changes the activity of recombinant PknG [38–40]; nitric oxide exposure, since fatty acid nitroalkenes react with the rubredoxin domain of recombinant PknG [41]; NADH since extracellular NADH induces PknG expression in *M*. *smegmatis* [11]. The influence of carbon sources on GarA phosphorylation (Fig 5) could potentially support the suggestion that PknG responds to redox balance or NADH, but these same carbon sources have also been found to influence the differential utilization of KDH or the alternative enzyme α-ketoglutarate ferredoxin oxidoreductase [42].

Several studies have highlighted the link between metabolic adaptation and pathogenicity, for example underlining the essentiality for intact central carbon metabolism and amino acid biosynthetic pathways for virulence [43–45]. The three enzymes controlled by PknG and GarA (KDH, GDH, GltS) have been mutated individually in independent studies [24–26]. Although comparison between different strains is complicated by potential differences in metabolism, disruption of *garA* might be expected to lead to glutamate auxotrophy like disruption of *gltS*, since glutamate synthase is activated by GarA [31], while disruption of *pknG* might be expected to lead to defects in glutamate catabolism, like disruption of *gdh* and *kdh*, since these enzymes are inhibited by GarA. The defects we observed in axenic culture match these predictions (Figs 1–3). However, unlike the *gltS* mutant, Δ*garA*_Mt_ had a severe defect in macrophages, probably reflecting the additional roles of GarA in inhibiting KDH and GDH. Mutants lacking KDH and GDH were attenuated in mice or macrophages, like *pknG* mutant or non-phosphorylatable *garA*. This could be due to their inability to utilise glutamate from host cells, but could also be due to increased susceptibility to stress as strains lacking KDH and GDH were reportedly more susceptible to killing by nitrosative stress [24, 25].

Apart from the role of PknG and GarA in regulating metabolism, other roles have been proposed, including regulating the cell envelope, antimicrobial resistance [12], stress resistance, biofilm formation [11], redox homeostasis [40], rhamnose biosynthesis [10] and glycogen metabolism [46]. While our focus has been on metabolism, our investigation has the potential to shed light on these alternative roles for PknG or GarA or to expose new roles. We saw evidence for important changes in amino acid metabolism when either *pknG* or *garA* were perturbed. Furthermore, in macrophages and in axenic culture there was phenotypic mimicry between disrupting *pknG* and disrupting the PknG phosphorylation site of GarA. Our data strongly suggest that the loss of virulence stems from loss of amino acid regulation. However, since unphosphorylated GarA can bind stably to STPKs, we can’t exclude non-physiological effects of GarA variants on other functions of PknG and other kinases. Indeed, since truncated GarA has been observed in cultured *M*. *tuberculosis* [47], GarA itself could influence kinase activity.

Prominent amongst the alternative functions of PknG are effects on the cell envelope and biofilm formation [[11, 12]. The clumping we observed in Δ*pknG*_Ms_ and *M*. *smegmatis* carrying non-phosphorylatable GarA could be linked to changes in the cell envelope. That would further suggest that the previously characterized changes in cell envelope or biofilm formation of *pknG* deficient strains could potentially be linked to the function of GarA in regulating metabolism. Regarding the other proposed functions of PknG and GarA in redox homeostasis and rhamnose biosynthesis, our metabolome data did not reveal significant changes in intracellular TDP-rhamnose, NAD+, NADH or FAD concentrations, but the changes in maltopentaose could potentially indicate changes in carbohydrate storage. It is possible that changes in the TCA cycle and carbon-nitrogen balance impact on other metabolic pathways, and also possible that disrupted kinase signaling could alter glycogen synthesis/breakdown directly since several of the enzymes are known to be regulated by phosphorylation [48].

Amongst all the proposed roles for PknG, only the function in regulating the TCA cycle has been investigated in other Actinobacteria, indeed this function was originally discovered in *C*. *glutamicum* [7]. Apart from the high level of interest afforded to a kinase linked to virulence, there are other differences between the PknG and GarA homologues between *M*. *tuberculosis* and *C*. *glutamicum*. While environmental/nutrient changes have been reported to influence phosphorylation of the GarA homologue in *C*. *glutamicum*, regulation of expression level is thought to play a major role [49]. By contrast, we did not observe increases in GarA expression in *M*. *tuberculosis* or *M*. *smegmatis* under the studied conditions where GarA was dephosphorylated (Figs 4 & 5). Thus, we conclude that the changing ratio of phosphorylated GarA to unphosphorylated GarA involves changes in kinase activity and/or phosphatase activity.

One of the paradigms of signaling by S/T phosphorylation in eukaryotes is that phosphorylation is reversible by serine/threonine protein phosphatases. Bacterial genomes encode S/T protein phosphatases, but kinetics of dephosphorylation have mainly been studied using recombinant proteins. Our observations of GarA phosphorylation in *M*. *smegmatis* showed that phosphorylation was a rapid response, occurring within minutes of exposure to amino acids, which is most likely due to stimulation of kinase activity. By contrast, removal of phosphorylated GarA during starvation occurred slowly over the course of several hours. This result suggests that dephosphorylation of GarA was very slow or may not have occurred at all, as dilution of GarA through cell division and protein turnover could account for the disappearance of the phosphorylated protein, while protein translation could account for the appearance of unphosphorylated GarA and this would remain unphosphorylated if PknG activity were low. The genome of *M*. *tuberculosis* encodes one S/T phosphatase, PstP (Rv0018c) compared to 11 STPKs, while *M*. *smegmatis* genome encodes two S/T phosphatases compared to 13 STPKs. PstP has been found to dephosphorylate recombinant GarA [50], which questions the value of *in vitro* methods for identification of phosphatase (and kinase) substrate specificity. Compared to kinases, even less is known of the physiological substrate specificity of phosphatases and their role(s). It remains to be seen whether the structure of GarA with self-binding of phosphothreonine by its own FHA domain [31, 50] makes it uniquely inaccessible to protein phosphatases or whether there are other bacterial phosphoproteins for which S/T phosphorylation in cells is effectively irreversible. The implication for the kinetics that we observed would be slow adaptation to starvation, requiring new GarA synthesis to inhibit GDH and KDH, but rapid adaptation to re-start the TCA cycle when starved/dormant cells were exposed to amino acids (Fig 8). This process of adaptation to non-growth and re-growth may be critical for survival of *M*. *tuberculosis in vivo* and may open new avenues for targeting non-growing bacteria that are notoriously tolerant to antimicrobials.

## Materials and methods

### Bacterial strains, media, and culture

*M*. *tuberculosis H37Rv* and *M*. *smegmatis* mc^2^155 were routinely cultured on Middlebrook 7H10 agar (Oxoid) with 10% ADN (0.5% bovine serum albumin, 0.2% dextrose, 0.085% NaCl) and Middlebrook 7H9 medium (Oxoid) with 10% ADN and 0.05% Tween 80. A list of strains and plasmids used in this study is provided (S8 & S9 Tables). To analyse nutrient utilization a minimal version of Sauton’s medium was made (3.7 mM KH_2_PO_4_, 2 mM MgSO_4_, 9.5 mM sodium citrate, 0.17 μM ferric ammonium citrate, pH 7.0 [51]), to which nutrients were added. Standard Sauton’s consisted of the recipe above with additional 1% glycerol, 30 mM asparagine and 0.05% Tween 80. To disperse the culture surfactants were added at 0.05% w/v: either Tween 80, which can be utilized as a carbon source, or tyloxapol, which cannot. When required antibiotics were used at the following concentrations: kanamycin (50 μg/mL), hygromycin (100 μg/ml).

### Deletion of *garA* from *M*. *tuberculosis H37Rv* to generate Δ*garA*_Mt_

We have previously characterized an *M*. *tuberculosis garA* conditional knockdown mutant (cΔ*garA*_Mtb_) in which the only functional copy of *garA* was inserted at the L5 att site under the transcriptional control of the repressible Pptr promoter using an L5-based integrative plasmid [9]. To obtain a *garA* null mutant, we decided to replace this plasmid with another one not containing *garA*. This is possible since introduction of an L5-integrative plasmid to a mycobacterial strain in which the L5 att site is already occupied by a similar plasmid leads to an efficient switching between the two plasmids [52]. To this end conditional mutant cΔ*garA*_Mtb_ was electroporated with pMV306, encoding hygromycin resistance, and switched mutants were selected on hygromycin (50 μg/ml) and asparagine (10 mM) to allow the growth of the *garA* null mutants. Hygromycin-resistant colonies were analysed to confirm the loss of kanamycin resistance (encoded in the *garA* containing integrative plasmid) and inability to grow in the absence of asparagine of the deletion strain Δ*garA*_Mt_.

### Plasmid complementation of Δ*garA*_Ms_ and Δ*garA*_Mt_ with variants of GarA

*M*. *smegmatis garA* with its promoter region was cloned in the plasmid pRBexint [53] to create pRBexint-*garA* as described [9]. *M*. *tuberculosis garA* was cloned with a hexahistidine tag into pRBexint to create pRBexint-His_6_*garA* and with a HA tag into pTTP1B [54] to create pTTP1B-*garA*HA. Variants of each gene were created by site directed mutagenesis to disrupt the phosphorylation motif ETTS. Also a truncated version of *M*. *smegmatis garA* was constructed, *garA*_39-143_, with the first 38 residues missing.

### Macrophage infection

THP-1 human cell line was grown at 37°C in a 5% CO_2_ atmosphere and maintained in RPMI medium (Gibco) supplemented with 10% fetal bovine serum (Gibco). After expansion, THP-1 cells were differentiated into macrophages and infected with *M*. *tuberculosis* in 96-well plates with a multiplicity of infection of 1:20 CFU per macrophage as previously described [55]. After 90 minutes of incubation at 37°C, the medium was removed, and cells were washed twice with 100 μl of warm phosphate buffered saline to remove extracellular bacteria. Finally, 100 μl of warm RPMI (with added 20 mM asparagine for S2 Fig), was added to each well and the plate was incubated at 37°C. RPMI with or without additional asparagine was replaced every 48 hours. To enumerate intracellular bacteria the medium was removed from three wells, and 100 μl of 0.05% sodium dodecyl sulfate was used to lyse macrophages. The suspensions obtained were immediately diluted in 7H9 and plated to determine viable counts. About 95% of macrophages remained viable during the entire experiment, as determined by Trypan blue exclusion.

### Ethics statement

All investigations involving animals were carried out according to the requirements of the Animals (Scientific Procedures) Act 1986 with the consent of the University of Leicester Animal Welfare & Ethics Board. The Home Office Licence number is 60/4327.

### Assessment of virulence in mice

BALB/c mice (female, 6–8 weeks old) were purchased from Charles River, UK and acclimatised for 7 days prior to *M*. *tuberculosis* challenge. Frozen aliquots of bacterial strains were thawed and passed through a blunt needle 10 times to disperse clumps and adjusted to 2x 10^6^ CFU ml^-1^ prior to infection. Mice were inoculated via the intranasal route by the drop-wise administration of 50 μl bacterial suspension onto the nostril of a lightly anaesthetised mouse (2.5% (v/v) flurothane over oxygen) held in a vertical position. Mice were monitored for full recovery from anaesthetic prior to return to their cages. Mice were housed in cages of 5 animals within a negative pressure rigid isolator (air change rate 25 changes/hr; pressure -100Pa). Mice had free access to water and diet (5LF2, LabDiet) and monitored daily for welfare and signs of disease over the 28-day experimental period.

Experimental groups (n = 20) were inoculated with either *M*. *tuberculosis H37Rv* wild type, Δ*garA*_Mt_ deletion or complementation strain at 10^5^ CFU per animal. A cage of 5 mice from each group was euthanised by cervical dislocation at day 1, 7, 21 and 28 and the lungs and spleen were aseptically removed post-mortem (conformation of death by rigor mortis). Lung and spleen tissue (lung only at day 1) was homogenised using a FastPrep-24 (MP Biomedicals) in 15ml tubes containing 10 matrix S beads (MP Biomedicals) and 9 ml PBS. Three bursts of 20 seconds at 4 m/s with a five minute cool-down in-between was used to homogenate the organs for enumeration of bacteria on 7H10 agar; kanamycin (50 μg/ml) and/or hygromycin (100 μg/ml) was added as required.

### Growth of *M*. *tuberculosis garA* deletion strain on Middlebrook 7H10 to examine the phenotype of *garA* gene deletion and complementation

*M*. *tuberculosis H37Rv* strains were grown in 7H9/ADN/Tween 80 with 30 mM asparagine until OD reached 0.6–0.9. The cultures were then diluted in Middlebrook 7H9 medium and plated on Middlebrook 7H10 ADN supplemented with and without 30 mM asparagine. Plates were incubated at 37°C and images taken after two weeks.

### Plasmid complementation of Δ*pknG*_Mt_ and growth in minimum Sauton’s medium

The gene encoding PknG was amplified by Pfu Ultra Hf DNA polymerase (Agilent) using an upper primer (AC 145) designed to contain an NheI site immediately before the start codon, and a lower primer (AC146) designed to contain the HA-coding sequence in frame with the coding sequence of *pknG*, followed by a stop codon and a XbaI site (S9 Table). For expression in *M*. *tuberculosis*, fragments containing the Phsp60 promoter sequence from HindIII/NheI-digested pAL36 [56] and the PknG-HA encoding gene digested with NheI/XbaI were transferred to HindIII/XbaI-digested pMV306 vector [57] giving the mycobacterial plasmid pAL299.

The plasmid was electroporated into strain Δ*pknG*_Mt_ and kanamycin-resistant recombinants that had integrated the vector with the PknG insert at the *attB* site were selected on Middlebrook 7H11-OADC (BD) plates and subjected to PCR screening and Western blotting. This approach allowed one colony to be selected that showed the expected PCR amplification products as well as the expected band in PknG-specific Western blotting (S7 Fig) (anti-PknG serum was a generous gift from E Houben and J Pieters, VU Medical Center, Amsterdam and University of Basel). This clone, “Δ*pknG*_Mt_ + *pknG*” was used for further experiments to evaluate the growth under selected amino-acid deprivation conditions.

In these experiments, the growth of *M*. *tuberculosis* wild-type, deletion and complemented strains was measured by monitoring OD_600_ of cultures grown in glass tubes at 37°C in standing conditions. Bacteria were grown until late-exponential phase (OD_600_ 0.6–0.9) in 7H9 ADC medium, washed twice and diluted in the Sauton’s minimum medium supplemented by a specific carbon source to an initial OD_600_ of 0.04–0.05. Sauton’s minimum medium was supplemented with ammonium chloride 10 mM plus one of the following carbon sources: L-Asparagine 10 mM, L-Glutamate 10 mM, Glycerol 0.2%, Glucose 1%, Acetate 0.2%. Data plotted represent the mean and standard deviation of at least three independent experiments.

### Measurement of nutrient requirements of *M*. *smegmatis*

The growth of *M*. *smegmatis* was measured by monitoring OD of cultures grown in microplates at 37°C with shaking. The inoculum used was a late-exponential phase culture (OD_600_ 0.6–0.9) in 7H9 ADN medium, which was dispersed by passing through a needle and then diluted in the required medium to an initial OD of 0.01. Growth curves used at least five wells per strain and were performed in triplicate. Figures show the mean and standard deviation for a representative experiment.

### Growth of *M*. *smegmatis* to prepare cellular proteins for determination of GarA phosphorylation

In order to analyse the phosphorylation status of GarA in *M*. *smegmatis* mc^2^155, strains were grown in 7H9 medium to OD_600_ 0.6–0.9 then diluted into 10 ml of 7H9 without ADN (or variant Sauton’s medium where specified) to OD_600_ 0.01 in a 50 ml falcon tube. Cultures were grown with shaking at 37°C and cultures were harvested at OD_600_ 0.6–0.9 by centrifugation at 4°C. Cells were either lysed by sonication either directly in SDS sample buffer or by sonication in cold Tris-buffered saline pH 8.0 containing PhosSTOP Phosphatase Inhibitors (Roche) and cOmplete Protease Inhibitors (Roche) followed by centrifugation and addition of SDS sample buffer. Protein concentration was measured by using BCA Protein Assay Reagent (Pierce).

### Growth of *M*. *tuberculosis* and preparation of cellular proteins to determine GarA phosphorylation

*M*. *tuberculosis H37Rv* strains were grown in standard Sauton’s medium containing 0.05% Tween 80 to OD_600_ 0.6–0.9 and then diluted into 30 ml of the specified Sauton’s medium to OD_600_ 0.01. Cultures were grown with shaking at 37°C to OD_600_ 0.6–1.0 and harvested by centrifugation. Cell pellets were resuspended in 1 ml SDS sample buffer (for mass spectrometry) or in 1 ml 1 M Tris HCl pH 8.0 containing PhosSTOP (Roche) and cOmplete (Roche) (for Phos-Tag analysis). Cells were lysed using a FastPrep (MP Biomedicals) with addition of glass beads 150–210 μm (Sigma) and insoluble material was removed by centrifugation. Cells were either killed by heating to 100°C for 30 minutes (for mass spectrometry) or were rendered non-infectious by filtration (for Phos-Tag). Protein concentration was measured by using BCA Protein Assay Reagent (Pierce).

### Determination of GarA phosphorylation by Western blotting using Phos-tag

7.5 μg protein was separated by SDS PAGE (10% acrylamide) containing 50 mM acrylamide-pendant Phos-tag ligand (Wako Pure Chemical) and 100 mM of Zn(NO_3_)_2_. The running buffer (pH 7.8) contained 0.1 M Tris HCl, 0.1 M 3-(N-morpholino) propanesulfonic acid (MOPS), 0.1% w/v SDS and 5 mM sodium bisulfite. The gel was washed as described previously [58] before transfer onto nitrocellulose membrane (Hybond-C extra, Amersham Biotech). Rabbit anti-GarA serum was kindly provided by Dr I Rosenkrands (Statens Serum Institute) and GarA was detected using goat anti-rabbit alkaline phosphatase (Sigma) with SIGMAFAST BCIP/NBT (Sigma). Blots were photographed and images analysed using ImageJ 1.45s software (U.S. National Institutes of Health, Bethesda, MD, USA) [59]. For SDS PAGE and blotting without Phos-tag ligand, the same serum, antibodies and method of analysis were used, as previously [9], and representative images are supplied (S17 Fig).

### Determination of GarA phosphorylation by mass spectrometry

Synthetic peptides corresponding to the tryptic peptides of GarA were purchased from CBio and analysed by LC-MS/MS using an RSLCnano HPLC system (Dionex) and 4000 Q-trap mass spectrometer (Applied Biosystems, Warrington, UK). Samples were loaded at high flow rate onto a reverse-phase trap column (0.3 mm i.d. x 1 mm), containing 5 μm C18 300 Å Acclaim PepMap media (Dionex) maintained at a temperature of 37°C. The loading buffer was 0.1% formic acid / 0.05% trifluoroacetic acid / 2% acetonitrile. After a 4 minute wash step, peptides were eluted from the trap column at the flow rate of 0.3 μl/min using an increasing proportion of mobile phase B (80% acetonitrile/0.1% formic acid); 4–45% B in 26 minutes, 45–90% B in 1 minute, held at 90% for 8 minutes, 90–4% in 1 minute, re-equilibration at 4% for 10 minutes. Eluted peptides were separated through a reverse-phase capillary column (75 μm i.d. x 250 mm) containing Symmetry C18 100 Å media (Waters) that was manufactured in-house using a high-pressure packing device (Proxeon Biosystems). The output from the column was sprayed directly into the nanospray ion source of the 4000 Q-Trap mass spectrometer.

The three forms of the peptide containing the phosphorylation sites (ETTS) and a control peptide (corresponding to a different tryptic peptide from the GarA protein) were found to separate by retention time and be distinguishable by their fragmentation spectra (Table 2). Multiple reaction monitoring (MRM) precursor/product ion transitions were chosen based on the fragmentation data and were used to produce standard curves for each peptide by injecting known amounts: 50, 100, 200, 400, 600 and 2000 fmol (S9 Fig).

**Table 2:** Precursor/Product ion transition used for multiple reaction monitoring of GarA peptides.

Peptide	Precursor Ion m/z (Da)	Product Ion m/z (Da)	Fragment type	Collision Energy (V)
DQTSDEVTVETTSVFR	907.43 (2+)	839.4	y_7_	47.9
		1039.5	y_9_	
DQTSDEVTVE**T**TSVFR^a^	947.41 (2+)	609.3	y_5_	49.5
		1021.5	y_9_-H_3_PO_4_	
		1119.5	y_9_	
DQTSDEVTVET**T**SVFR^a^	947.41 (2+)	591.3	y_5_-H_3_PO_4_	49.5
		1021.5	y_9_-H_3_PO_4_	
		1119.5	y_9_	
EPVDSAVLANGDEVQIGK	920.97 (2+)	1030.5	y_10_	42.0
		1143.6	y_11_	

^a^Phosphothreonine is indicated in bold.

Soluble protein extracts of *M*. *tuberculosis* were run on 1D-gels, the region of interest excised, and in-gel trypsin digestion carried out upon each. Gel slices was destained using 200 mM ammonium bicarbonate/20% acetonitrile, followed by reduction (10 mM dithiothreitol, Melford Laboratories Ltd., Suffolk, UK), alkylation (100 mM iodoacetamide, Sigma, Dorset, UK) and enzymatic digestion with trypsin (sequencing grade modified porcine trypsin, Promega, Southampton, UK) in 50 mM triethylammonium bicarbonate (Sigma) using an automated digest robot (Multiprobe II Plus EX, Perkin Elmer, UK). After overnight digestion, samples were acidified using formic acid (final concentration 0.1%) and analysed by LC-MS/MS using the gradient and MRM transitions outlined above. At the start and end of each analytical run, the synthetic peptides were analysed to allow comparison with the standard curves for the purpose of assessing technical reproducibility.

### PBS starvation and extended stationary phase of *M*. *smegmatis*

For the PBS starvation experiment using *M*. *smegmatis*, strains were cultured and harvested as described above. Pellets were washed twice in PBS and resuspended in 30 ml PBS with 0.05% tyloxapol and incubated at 37°C without shaking for up to 5 days. Samples were taken at different time points for analysis of GarA phosphorylation. For analysis of GarA during stationary phase, cells were cultured as described above and incubated for 5 days at 37°C with shaking. For the extended stationary phase experiment strains were cultured from single colonies in 5 ml 7H9/ADN/Tween 80 in a closed 30 ml Universal tube for 3 months at 37°C with shaking. The first sample was taken after the culture reached late exponential phase (OD_600_ 0.6–1.0) and bacterial viability was estimated by measuring CFU ml^-1^ by plating aliquots of bacterial suspension on 7H10/ADN plates. Further samples were taken every 4 weeks and CFU determined.

### Metabolomics

Samples for metabolic analysis were collected during early exponential growth phase (OD_600_ 0.3–0.5) by fast filtration as described previously for Mycobacteria [60]. Briefly, a sample volume equivalent to a biomass of 4 ml at OD_600_ of 1.0 was filtered (MF-Millipore Membrane, 0.45 μm), briefly washed with ammonium carbonate buffer (75 mM, pH 6.6) and transferred to 3 ml ethanol 60% (v/v) at 78°C for 2 min. Samples were dried at 30°C in a SpeedVac equipped with a cooling trap at -85°C. The dried extracts were dissolved in 100 μl water for metabolite analysis. Sample collection and processing of extracellular samples for metabolomics analysis was performed as previously described [61]. Quantification by targeted mass spectrometry was performed by ion pairing–reverse phase liquid chromatography tandem mass spectrometry on a Waters Acquity UHPLC coupled to a Thermo TSQ Quantum Ultra triple quadrupole instrument using fully U-^13^C-labled yeast extract as internal standard [62]. Non-targeted mass spectrometry was performed on an Agilent 6550 QTOF instrument [63]. Annotation was performed based on accurate mass determination of ions and the KEGG reference list (tolerance 0.001 Da). Removal of unknown ions and annotated ion adducts resulted in 397 putatively annotated ions with unique m/z. Two technical replicate measurements were performed for each sample and merged using their mean.

Differential analysis was performed applying an unequal t-test using MatLab (The Mathwork, Natick). For each metabolite, pairwise comparisons were made between test strain and wild type using an unequal t-test, resulting in fold-change and associated q-value (corrected for multiple hypotheses using the Benjamini Hochberg procedure). The results of pairwise comparisons are graphically represented as volcano plots. We chose criteria for significant changes of q<0.05 and absolute log2(fold change)>0.5. The borders are shown in the volcano plots and metabolites passing these thresholds are coloured red if higher than wild type or blue if lower. The metabolites scored as significant were compiled for each strain and these lists used for comparison between multiple strains. Metabolites that were significantly changed in >1 strain are highlighted by asterisks in the tables and fold-changes in concentration compared to wild type are summarized in Fig 7D. Some metabolites, including cAMP and mycobactin, were significantly altered in most strains, including complemented strains and other mutant strains unrelated to this project. It is possible that there were technical reasons making the extraction or quantification of these metabolites more variable, or alternatively these metabolite pools may be more susceptible to changes caused by cell stress. These variable metabolites were excluded from Table 1 and are listed in S1 Table.

## Supporting information

S1 Table Intracellular metabolites that were at lower concentration in Δ*garA*_Ms_ than wild type *M*. *smegmatis*. (DOCX)

S2 Table Intracellular metabolites that were at higher concentration in both strains of Δ*garA*_Ms_ carrying non-phosphorylatable GarA than in wild type. (DOCX)

S3 Table Intracellular metabolites that were at lower concentration in both strains of Δ*garA*_Ms_ carrying non-phosphorylatable GarA than in wild type. (DOCX)

S4 Table Intracellular metabolites that were at higher concentration in Δ*pknG*_Ms_ than in wild type. (DOCX)

S5 Table Intracellular metabolites that were at lower concentration in Δ*pknG*_Ms_ than in wild type. (DOCX)

S6 Table Fold-change and q-values for intracellular metabolite concentrations comparing strains of *M*. *smegmatis*. (XLSX)

S7 Table Fold-change and q-values for intracellular metabolite concentrations comparing *pknG*-disrupted *M*. *bovis* BCG to the parental strain. (XLSX)

S8 Table Strains and plasmids. (DOCX)

S9 Table Oligonucleotides. (DOCX)

S1 Fig Δ*garA*_Mt_ grew poorly in Middlebrook 7H9 broth, but growth was partially restored by supplementation with 10 mM asparagine and fully restored by re-introduction of *garA*. *M*. *tuberculosis* lacking *garA* (red squares) had a defect in growth in Middlebrook 7H9 broth compared to parental *M*. *tuberculosis H37Rv* (black circles). Extracellular asparagine (10 mM) partially restored the growth of Δ*garA*_Mt_ (red triangles with dashed line), while re-introduction of *garA* (black triangles) fully restored growth. Data points show the mean and standard deviation from 3 independent replicates.
(TIFF)

S2 Fig Addition of 10 mM asparagine to the growth medium did not restore the growth defect of Δ*garA*_Mt_ in THP-1 macrophages. *M*. *tuberculosis* lacking *garA* (red squares) had a defect in growth and survival in differentiated THP-1 cells compared to parental *M*. *tuberculosis H37Rv* (black circles). Extracellular asparagine (20 mM) did not restore the growth of Δ*garA*_Mt_ (red triangles with dashed line). Data points show the mean and standard deviation from 4 replicates and are representative of two independent experiments.
(TIFF)

S3 Fig Plasmid encoded GarA and ETTS-motif variants were expressed in Δ*garA*_Mt_. *M*. *tuberculosis* cell extract was analysed by Western blot probed with anti-GarA antibody. Loading was normalised by SDS PAGE and Coomassie staining. Native GarA has higher mobility than FLAG-tagged GarA encoded on the plasmids.
(TIFF)

S4 Fig Δ*pknG*_Mt_ replicated more slowly than parental *M*. *tuberculosis* H37Rv in THP-1 macrophages. *M*. *tuberculosis* lacking *pknG* (grey bars) had a defect in growth in differentiated THP1 cells compared to parental *M*. *tuberculosis H37Rv* (black bars). Data points show the mean and standard deviation of two independent biological replicates with three technical replicates. **p<0.01 student’s t test.
(TIFF)

S5 Fig The growth phenotypes measured in microplates were also observed in flasks. *M*. *smegmatis garA* mutant grew poorly on propionate, while *M*. *smegmatis pknG* mutant grew poorly and formed clumps when glutamate was the sole nitrogen source.
(TIFF)

S6 Fig Δ*pknG*_Mt_ grew at a similar rate to parental *M*. *tuberculosis* in minimum medium containing glucose or acetate as the sole carbon source. Alternative carbon sources were tested to characterise the nutrient-dependent growth defect of Δ*pknG*_Mt_. The *pknG* deficient strain had a growth defect only when glutamate or asparagine were the sole carbon source in minimal medium (main manuscript Fig 3). When other carbon sources were used of Δ*pknG*_Mt_ (blue diamonds) grew at the same rate as the parent strain (black circles). Glucose or acetate were added to minimal Sauton’s medium at 0.2%. Graphs show measurements from a single experiment that is representative of multiple independent experiments.
(TIFF)

S7 Fig Plasmid pAL299 restored expression of PknG expression in *ΔpknG*_Mt_. *M*. *tuberculosis* H37Rv (1), Δ*pknG*_Mt_ (2) and Δ*pknG*_Mt_ + pAL299 (3) whole cell lysates were analysed by western blotting. Equal amount of cell equivalents were loaded and proteins were detected using anti-PknG serum diluted 1:4000, anti SigA serum diluted 1:4000 and HRP-anti rabbit antibody.
(TIFF)

S8 Fig *M*. *tuberculosis pknG* and *garA* restored the growth defects of the equivalent *M*. *smegmatis* mutant strains. (A) *M*. *tuberculosis pknG* was introduced into Δ*pknG*_Ms_ leading to restoration of the ability to grow on minimal Sauton’s medium containing 10 mM glutamate, 1% glycerol and 0.05% tween 80 as sole carbon/nitrogen sources. (B) *M*. *tuberculosis garA* was introduced into Δ*garA*_Ms_ leading to partial restoration of the ability to grow on minimal Sauton’s medium containing 20 mM propionate and 10 mM NH_4_Cl as sole carbon/nitrogen source with 0.05% tyloxapol to prevent clumping. The growth experiment shown is a representative of three independent experiments. Data plotted are mean and standard deviation of three technical replicates and are representative of 3 independent experiments.
(TIFF)

S9 Fig Synthetic GarA peptide standards were analysed by LC-MS/MS. In preparation for analysis of tryptic peptides of GarA (main Fig 4), equivalent synthetic peptides were analysed by LC-MS/MS as a mix in 1:1:1 molar ratio (**A**) or singly at a range of concentrations (**B-D**).
(TIFF)

S10 Fig Phosphorylated GarA was detected in cell extracts of *M*. *tuberculosis* by LC-MS/MS. The tryptic peptide corresponding to T21-phosphorylated GarA (EpTTS) was absent from extracts of Δ*pknG*_Mt_ suggesting that PknG is the main kinase responsible for phosphorylating GarA.
(TIFF)

S11 Fig Hexahistidine tagged GarA complemented the growth defect of Δ*garA*_Ms_. Hexahistidine-tagged GarA can complement the growth defect of Δ*garA*_Ms_. Growth curves of *M*. *smegmatis* mc^2^155, Δ*garA*_Ms_, and complemented Δ*garA*_Ms_ strain with His_6_ tag in standard Sauton’s medium (A) or modified Sauton’s (B) containing 20 mM propionate, tyloxapol and 10 mM NH4Cl. The growth experiment shown is a representative of three independent experiments. Data plotted are mean and standard deviation of five technical replicates.
(TIFF)

S12 Fig In PBS-starved *M*. *tuberculosis* H37Rv phosphorylated GarA was reduced compared to unphosphorylated GarA as detected by mass spectrometry. Graphs are representative of at least 3 independent experiments.
(TIFF)

S13 Fig Dephosphorylation of GarA. To verify that loss of GarA phosphorylation was related to change of nutrients rather than cessation of growth, *M*. *smegmatis* was maintained in phosphorylation medium (black bars show % of GarA phosphorylated and black line shows optical density) or switched to minimal medium at t = 0 (grey bars show % phosphorylation and grey line shows optical density) and optical density was monitored in parallel to sampling for phosphorylation analysis.
(TIFF)

S14 Fig Intracellular metabolites from *M*. *smegmatis* in stationary phase. These measurements verify that the Δ*garA*_Ms_ cells had not lysed at day 28 (time point 6). The line represents the mean and shaded area shows standard deviation from 3 independent cultures.
(TIFF)

S15 Fig Extracellular metabolites from *M*. *smegmatis*. Extracellular metabolite concentrations of *M*. *smegmatis* mutant strains grown in Middlebrook 7H9 broth did not show significant changes compared to wild type.
(TIFF)

S16 Fig Intracellular metabolites from Δ*pknG*_Ms_ and the complemented strain. (**A**) *M*. *smegmatis* expressing unphosphorylatable GarA had altered intracellular metabolites compared to wild type. The changes were similar to those seen in the strain expressing truncated GarA (main text Fig 7C). (**B**) Δ*pknG*_Ms_ had an altered intracellular metabolome compared to wild type. (**C**) reintroduction of *pknG* partially restored the perturbations. All strains were grown in Middlebrook 7H9 broth.
(TIFF)

S17 Fig Western blots used to make Fig 5. Dashed lines mark the regions of the blots displayed in Fig 5. These images are representative of at least 3 samples for each condition that were analysed to calculate the ratios in Fig 5. The anti-GarA serum gave several non-specific bands, but His_6_-GarA was identified with confidence by comparison with molecular weight markers and by comparison with *garA*-deleted *M*. *smegmatis* (panel **F**).
(TIFF)
