# Supplementary material for: PknG senses amino acid availability to control metabolism and virulence of Mycobacterium tuberculosis
Source: PLoS Pathog. 2017 May 17;13(5):e1006399. doi: 10.1371/journal.ppat.1006399 (PMC5448819; doi:10.1371/journal.ppat.1006399)
Supplement: S1 Table — (DOCX) [file ppat.1006399.s001.docx]

**Table S1.** Intracellular metabolites that were at lower concentration in Δ*garA*_Ms_ than wild type *M. smegmatis* but restored in complemented Δ*garA*_Ms_ + *garA*. * denotes amino acid metabolism. ** denotes metabolites that are also significantly changed in Δ*pknG*_Ms_.

| Metabolite | Pathway | Fold change | q-value |
| --- | --- | --- | --- |
| 1D-myo-inositol/2-Acetamido-2-deoxy-D-glucopyranoside | Mycothiol biosynthesis | 0.455 | <0.001 |
| (E,E,E,E,E,E,E,Z,Z) decaprenyl phosphate | Cell wall synthesis | 0.476 | <0.001 |
| LL-2,6-Diaminoheptanedioate/Meso-2,6-Diaminoheptanedioate | * Lys biosynthesis | 0.485 | <0.001 |
| **O-Phospho-L-serine | * Ser biosynthesis | 0.505 | <0.001 |
| sn-Glycero-3-phosphoethanolamine | Lipid metabolism | 0.523 | <0.001 |
| Glutamate | * Amino acid | 0.555 | <0.001 |
| **4-Aminobutanoate | * GABA (from glu) | 0.570 | 0.003 |
| N2-Formyl-N1-(5-phospho-D-ribosyl)glycinamide | Purine metabolism | 0.612 | <0.001 |
| N-Pantothenoylcysteine | Pantothenate/CoA biosynthesis | 0.612 | 0.001 |
| N-Succinyl-2-L-amino-6-oxoheptanedioate | * Lys biosynthesis | 0.613 | <0.001 |
| Aspartate | * Amino acid | 0.629 | <0.001 |
| 5-Oxoproline | * Pro biosynthesis | 0.637 | <0.001 |
| Phosphatidylglycerophosphate (dituberculostearoyl, C19:0) | Glycerophospholipid metabolism | 0.646 | <0.001 |
| Serine | * Amino acid | 0.674 | 0.005 |
| D-Alanyl-D-alanine | Peptidoglycan biosynthesis | 0.706 | 0.012 |
