# Supplementary material for: PknG senses amino acid availability to control metabolism and virulence of Mycobacterium tuberculosis
Source: PLoS Pathog. 2017 May 17;13(5):e1006399. doi: 10.1371/journal.ppat.1006399 (PMC5448819; doi:10.1371/journal.ppat.1006399)
Supplement: S2 Table — (DOCX) [file ppat.1006399.s002.docx]

**Table S2.** Intracellular metabolites that were at higher concentration in both strains of Δ*garA*_Ms_ carrying non-phosphorylatable GarA than in wild type. * denotes amino acid metabolism. ** denotes metabolites also significantly changed in Δ*pknG*_Ms_.

| Metabolite | Pathway | Fold change  (trunc. GarA) | q-value | Fold change (EAAS GarA) | q-value |
| --- | --- | --- | --- | --- | --- |
| O-Acetyl-L-homoserine | *Cys/Met metabolism | 3.69 | <0.001 | 1.87 | 0.004 |
| Lactose/Maltose/Trehalose | Storage/osmolyte | 1.99 | <0.001 | 1.60 | <0.001 |
| Pyridoxal 5'-phosphate | Coenzyme for transaminations | 1.93 | <0.001 | 1.91 | <0.001 |
| N2-Acetyl-L-ornithine | *Arg biosynthesis | 1.91 | <0.001 | 1.88 | <0.001 |
| **Citrulline | *Arg biosynthesis | 1.73 | <0.001 | 1.69 | <0.001 |
| **Ornithine | *Arg biosynthesis | 1.62 | <0.001 | 1.57 | <0.001 |
| Phosphatidylethanolamine | Membrane | 1.62 | 0.004 | 1.52 | 0.004 |
| Proline | *Amino acid | 1.58 | <0.001 | 1.44 | <0.001 |
| FAD | Cofactor (redox) | 1.54 | <0.001 | 1.55 | <0.001 |
| Hydrogenobyrinate a,c diamide | Heme synthesis | 1.49 | 0.003 | 1.62 | 0.012 |
| Ribose phosphate isomers | Pentose phosphate pathway | 1.48 | <0.001 | 1.57 | <0.001 |
| Phenylacetaldehyde | *Phe metabolism | 1.45 | <0.001 | 1.59 | 0.003 |
