# Supplementary material for: PknG senses amino acid availability to control metabolism and virulence of Mycobacterium tuberculosis
Source: PLoS Pathog. 2017 May 17;13(5):e1006399. doi: 10.1371/journal.ppat.1006399 (PMC5448819; doi:10.1371/journal.ppat.1006399)
Supplement: S3 Table — (DOCX) [file ppat.1006399.s003.docx]

**Table S3.** Intracellular metabolites that were at lower concentration in both strains of Δ*garA*_Ms_ carrying non-phosphorylatable GarA than in wild type. * denotes amino acid metabolism. ** denotes metabolites also significantly changed in Δ*pknG*_Ms_.

| Metabolite | Pathway | Fold change  (trunc. GarA) | q-value | Fold change (EAAS GarA) | q-value |
| --- | --- | --- | --- | --- | --- |
| Thiocyanate | *Cys/Met metabolism | 0.442 | 0.008 | 0.468 | 0.012 |
| **Maltopentaose | Polysaccharide | 0.534 | <0.001 | 0.647 | 0.002 |
| Glucosamine phosphate (isomers) | *Amino acid/sugar metabolism | 0.551 | <0.001 | 0.685 | <0.001 |
| Riboflavin | Cofactor | 0.568 | <0.001 | 0.522 | <0.001 |
| 4-Amino-5-hydroxmethl-2-methylpyrimidine | Thiamine metabolism | 0.582 | <0.001 | 0.565 | <0.001 |
| **2,3-Dihydroxy-3-methylpentanoate/Pantoate | *Val/Leu/Ile biosynthesis | 0.623 | 0.013 | 0.525 | 0.005 |
| 2-Isopropylmaleate | *Val/Leu/Ile biosynthesis | 0.640 | 0.002 | 0.665 | 0.016 |
