# Supplementary material for: PknG senses amino acid availability to control metabolism and virulence of Mycobacterium tuberculosis
Source: PLoS Pathog. 2017 May 17;13(5):e1006399. doi: 10.1371/journal.ppat.1006399 (PMC5448819; doi:10.1371/journal.ppat.1006399)
Supplement: S4 Table — (DOCX) [file ppat.1006399.s004.docx]

**Table S4.** Intracellular metabolites that were at higher concentration in Δ*pknG*_Ms_ than in wild type. * denotes amino acid metabolism. ** denotes metabolites also significantly changed in Δ*garA*_Ms_ or in Δ*garA*_Ms_ with non-phosphorylatable GarA.

| Metabolite | Pathway | Fold change | q-value |
| --- | --- | --- | --- |
| **Ornithine | *Arg biosynthesis | 3.476 | <0.001 |
| **L-Citrulline | *Arg biosynthesis | 3.096 | <0.001 |
| GMP | Purine metabolism | 2.603 | <0.001 |
| **O-Phospho-L-serine | *Serine biosynthesis | 1.947 | 0.018 |
| GDP | Purine metabolism | 1.905 | 0.005 |
| Betaine aldehyde | *Gly/Ser/Thr metabolism | 1.866 | 0.041 |
| **(R)-2,3-Dihydroxy-3-methylpentanoate/(R)-Pantoate | *Val/Leu/Ile biosynthesis | 1.731 | 0.002 |
| Methyl octadecanoate/10-methylstearic acid | Lipid metabolism | 1.630 | 0.048 |
| IMP | Purine metabolism | 1.580 | 0.026 |
| Di-methyl behenic acid/Tetracosanoate (n-C24:0) | Lipid metabolism | 1.552 | 0.024 |
| **4-Aminobutanoate | *GABA from Glu | 1.544 | 0.005 |
| Dimethyl lignoceric acid/Hexacosanoate (n-C26:0) | Lipid metabolism | 1.399 | 0.042 |
| 3-Phosphohydroxypyruvate | *Multiple | 1.390 | 0.027 |
