# Supplementary material for: PknG senses amino acid availability to control metabolism and virulence of Mycobacterium tuberculosis
Source: PLoS Pathog. 2017 May 17;13(5):e1006399. doi: 10.1371/journal.ppat.1006399 (PMC5448819; doi:10.1371/journal.ppat.1006399)
Supplement: S5 Table — (DOCX) [file ppat.1006399.s005.docx]

**Table S5.** Intracellular metabolites that were at lower concentration in Δ*pknG*_Ms_ than in wild type. * denotes amino acid metabolism. ** denotes metabolites also significantly changed in Δ*garA*_Ms_ with non-phosphorylatable GarA.

| Metabolite | Pathway | Fold change | q-value |
| --- | --- | --- | --- |
| 2-C-methyl-D-erythritol 4-phosphate | Isoprenoid biosynthesis | 0.055 | 0.016 |
| Glycerol | Multiple | 0.163 | 0.013 |
| Citrate/Isocitrate | TCA cycle | 0.392 | 0.015 |
| N-Carbamoyl-L-aspartate | * Ser biosynthesis | 0.444 | 0.002 |
| N2-Succinyl-L-glutamate | * Arg metabolism | 0.449 | 0.034 |
| L-Aspartate | * Amino acid | 0.473 | 0.002 |
| Coenzyme A | Cofactor | 0.582 | 0.020 |
| Gamma-L-glutamyl-L-cysteine | Glutathione metabolism | 0.652 | 0.023 |
| Dihydroorotate | Pyrimidine metabolism | 0.653 | 0.009 |
| Alanine | * Amino acid | 0.656 | 0.015 |
| **Maltopentaose | Polysaccharide | 0.661 | 0.036 |
| L-Aspartate 4-semialdehyde | * Amino acid metabolism | 0.697 | 0.031 |

Full datasets for comparisons of intracellular metabolite concentrations are provided as separate excel files (table S6 and S7).
