# Supplementary material for: PknG senses amino acid availability to control metabolism and virulence of Mycobacterium tuberculosis
Source: PLoS Pathog. 2017 May 17;13(5):e1006399. doi: 10.1371/journal.ppat.1006399 (PMC5448819; doi:10.1371/journal.ppat.1006399)
Supplement: S8 Table — (DOCX) [file ppat.1006399.s008.docx]

Table S8. Bacterial strains and plasmids used in this study

| **Strain and plasmid** | **relevant characteristics** | **Source or reference** |
| --- | --- | --- |
| *M. smegmatis* mc^2^155 | wild type strain | ATCC700084 |
| Δ*pknG*_MS_ | *pknG* disrupted *M. smegmatis* | ([Wolff *et al.*, 2009](#_ENREF_3)) |
| Δ*pknG*_MS_ + *pknG* | Δ*pknG*_MS_ transformed with pVN579 to express *pknG* from heat shock promoter | ([Wolff *et al*., 2009](#_ENREF_3)) |
| *∆garA*_MS_ | *garA* disrupted *M. smegmatis* | ([Ventura *et al.*, 2013](#_ENREF_2)) |
| *∆garA*_MS_  + *garA* | *∆garA*_MS_ transformed with pRB*garA*; *garA* expressed from own promoter | ([Ventura *et al*., 2013](#_ENREF_2)) |
| *∆garA*_MS_ + *garA* trunc. | *∆garA*_MS_ transformed with pRB*garA*_39-143_ to express truncated GarA | this study |
| *∆garA*_MS_ + *garA* EAAS | *∆garA*_MS_ transformed with pRB*garA* EAAS: “non-phosphorylatable GarA” | this study |
| Reporter strain *∆garA*_MS_ + *his*_6_*garA* | *∆garA*_MS_ transformed with pRB*his*_6_*garA* to express hexahistidine-GarA | this study |
| *∆garA*_MS_ + *his*_6_*garA* EATS | *∆garA*_MS_ transformed with variants of pRB*his*_6_*garA* with disrupted phosphorylation sites | this study |
| *∆garA*_MS_ + *his*_6_*garA* ETAS |  | this study |
| *∆garA*_MS_ + *his*_6_*garA* EAAS |  | this study |
| *M. tuberculosis* H37Rv | wild type strain | ([Cowley *et al.*, 2004](#_ENREF_1)) |
| Δ*pknG*_Mt_ | *pknG* disrupted *M. tuberculosis* | ([Cowley *et al*., 2004](#_ENREF_1)) |
| Δ*pknG*_Mt_ pAL299 | Δ*pknG*_Mt_ with pMV306-*phsp60*-*pknG-HA* | this study |
| Δ*pknG*_BCG_ | *pknG* disrupted *M. bovis* BCG | (Walburger *et al*., 2004) |
| *M. bovis BCG* (Pasteur) | parental *M. bovis* BCG | (Walburger *et al*., 2004) |
| c*∆garA*_Mt_ | conditional *garA* mutant of *M. tuberculosis* used to construct *∆garA*_Mt_ | (Ventura *et al*., 2013) |
| *∆garA*_Mt_ | *garA* disrupted *M. tuberculosis* | this study |
| *∆garA*_Mt_ + *garA* | *∆garA*_Mt_ with plasmid pTTP1B*garA* | this study |
| *∆garA*_Mt_ + *garA* EATS | *∆garA*_Mt_ with plasmid pTTP1B*garA* with mutations at the ETTS phosphorylation motif | this study |
| *∆garA*_Mt_ + *garA* ETAS |  | this study |
| *∆garA*_Mt_ + *garA* EAAS |  | this study |

ATCC: American Type Culture Collection.

Cowley, S., M. Ko, N. Pick, R. Chow, K.J. Downing, B.G. Gordhan, J.C. Betts, V. Mizrahi, D.A. Smith, R.W. Stokes & Y. Av-Gay, (2004) The Mycobacterium tuberculosis protein serine/threonine kinase PknG is linked to cellular glutamate/glutamine levels and is important for growth in vivo. *Molecular Microbiology* **52**: 1691-1702.

Ventura, M., B. Rieck, F. Boldrin, G. Degiacomi, M. Bellinzoni, N. Barilone, F. Alzaidi, P.M. Alzari, R. Manganelli & H.M. O’Hare, (2013) GarA is an essential regulator of metabolism in *Mycobacterium tuberculosis*. *Mol Micro*.

Walburger, A., A. Koul, G. Ferrari, L. Nguyen, C. Prescianotto-Baschong, K. Huygen, B. Klebl, C. Thompson, G. Bacher & J. Pieters, (2004) Protein kinase G from pathogenic mycobacteria promotes survival within macrophages. *Science* **304**: 1800-1804.

Wolff, K.A., H.T. Nguyen, R.H. Cartabuke, A. Singh, S. Ogwang & L. Nguyen, (2009) Protein kinase G is required for intrinsic antibiotic resistance in mycobacteria. *Antimicrobial agents and chemotherapy* **53**: 3515-3519.
