# Supplementary material for: PknG senses amino acid availability to control metabolism and virulence of Mycobacterium tuberculosis
Source: PLoS Pathog. 2017 May 17;13(5):e1006399. doi: 10.1371/journal.ppat.1006399 (PMC5448819; doi:10.1371/journal.ppat.1006399)
Supplement: S9 Table — (DOCX) [file ppat.1006399.s009.docx]

Table S9. Oligonucleotides 5’-3’

MS_GarAT21A_F

AAGACCGATGTGGCCTCTACCGTCACATCC

MS_GarAT21A_R

GACGGTAGAGGCCACATCGGTCTTCCGCGC

MS_GarAT22A_F

AAGACCGATGCGGTCTCTACCGTCACATCC

MS_GarAT22A_R

GACGGTAGAGACCGCATCGGTCTTCCGCGC

MS_GarAT21A/T22A_F

AAGACCGATGCGGCCTCTACCGTCACATCC

MS_GarAT21A/T22A_R

GACGGTAGAGGCCGCATCGGTCTTCCGCGC

MS_GarAEcoRI__39-143_

ATGTGAATTCATGTCGGCTTTGCTTGTCGTCA

MS_GarAHisHpaI_F

GATCGTTAACTCAgtgatgatgatgatgGGCGTTCGAGCCGCTGTCG

RV_GarAT21A_F

GTAGAGGCGACCTCCGTCTTCCGCGCAGACTTCC

RV_GarAT21A_R

AGGTCGCCTCTACCGTGACTTCATCGGAGGTCTGG

RV_GarAT22A_F

GAGACGGCCTCCGTCTTCCGCGCAGACTTCCTCAGC

RV_GarAT22A_R

ACGGAGGCCGTCTCTACCGTGACTTCATCGGAGGTC

RV_GarAT21A/T22A_F

GTAGAGGCGGCCTCCGTCTTCCGCGCAGACTTCCTC

RV_GarAT21A/T22A_R

GGAGGCCGCCTCTACCGTGACTTCATCGGAGGTCTGG

AC145

ACGCTAGCATGGCCAAAGCG TCAGAG

AC146

ACTCTAGATTAAGCATAATCAGGAACATCATACGGATAGAACGTGCTGGTGGGCCGGACC
