# Supplementary figures and images for: PknG senses amino acid availability to control metabolism and virulence of Mycobacterium tuberculosis
Source: PLoS Pathog. 2017 May 17;13(5):e1006399. doi: 10.1371/journal.ppat.1006399 (PMC5448819; doi:10.1371/journal.ppat.1006399)

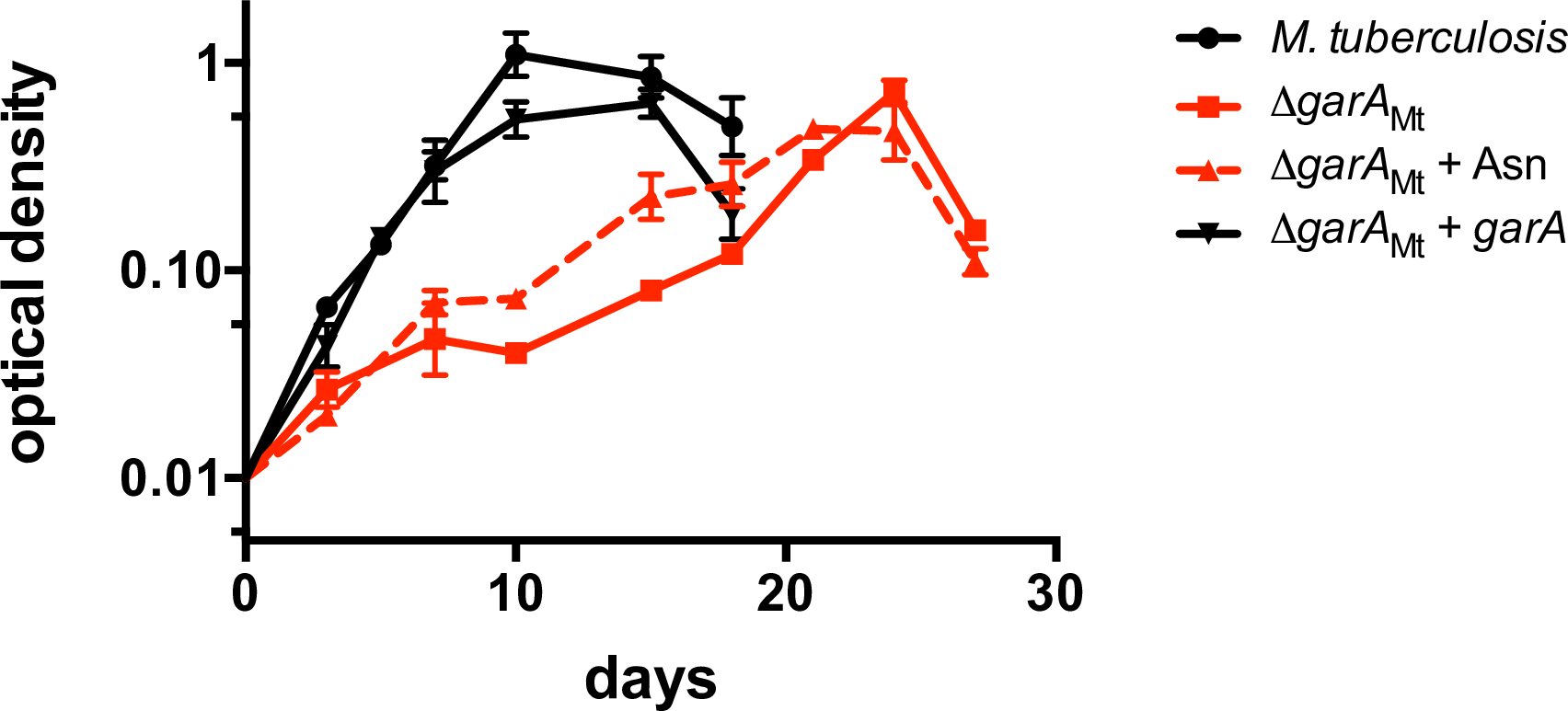

Supplement: S1 Fig — M. tuberculosis lacking garA (red squares) had a defect in growth in Middlebrook 7H9 broth compared to parental M. tuberculosis H37Rv (black circles). Extracellular asparagine (10 mM) partially restored the growth of ΔgarAMt (red triangles with dashed line), while re-introduction of garA (black triangles) fully restored growth. Data points show the mean and standard deviation from 3 independent replicates. (TIFF) [file ppat.1006399.s010.tiff]

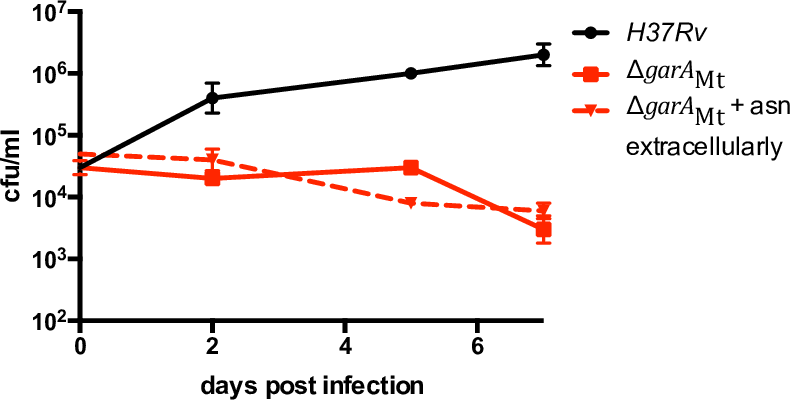

Supplement: S2 Fig — M. tuberculosis lacking garA (red squares) had a defect in growth and survival in differentiated THP-1 cells compared to parental M. tuberculosis H37Rv (black circles). Extracellular asparagine (20 mM) did not restore the growth of ΔgarAMt (red triangles with dashed line). Data points show the mean and standard deviation from 4 replicates and are representative of two independent experiments. (TIFF) [file ppat.1006399.s011.tiff]

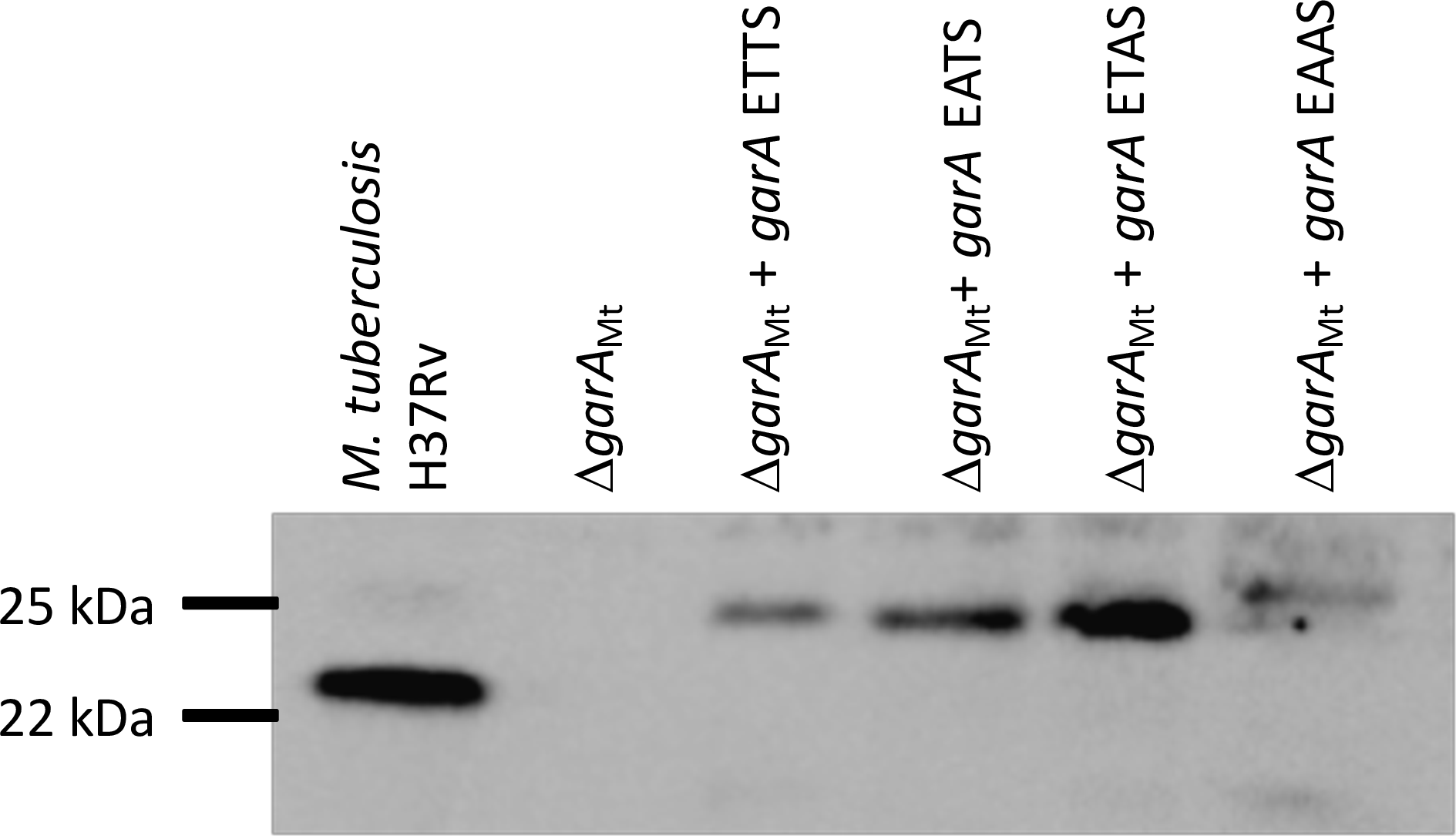

Supplement: S3 Fig — M. tuberculosis cell extract was analysed by Western blot probed with anti-GarA antibody. Loading was normalised by SDS PAGE and Coomassie staining. Native GarA has higher mobility than FLAG-tagged GarA encoded on the plasmids. (TIFF) [file ppat.1006399.s012.tiff]

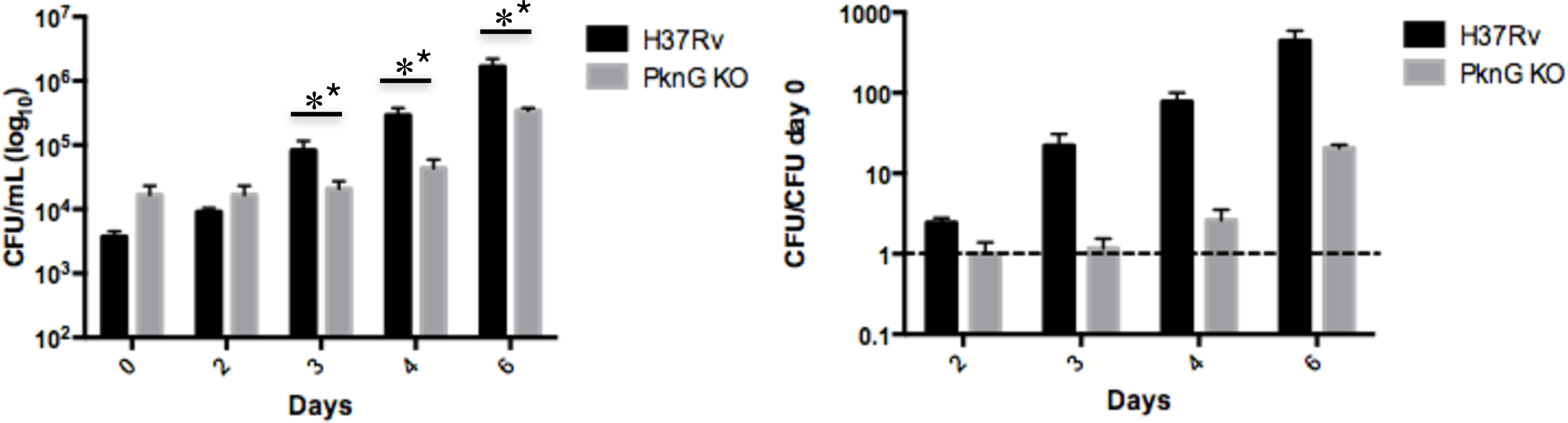

Supplement: S4 Fig — M. tuberculosis lacking pknG (grey bars) had a defect in growth in differentiated THP1 cells compared to parental M. tuberculosis H37Rv (black bars). Data points show the mean and standard deviation of two independent biological replicates with three technical replicates. **p<0.01 student’s t test. (TIFF) [file ppat.1006399.s013.tiff]

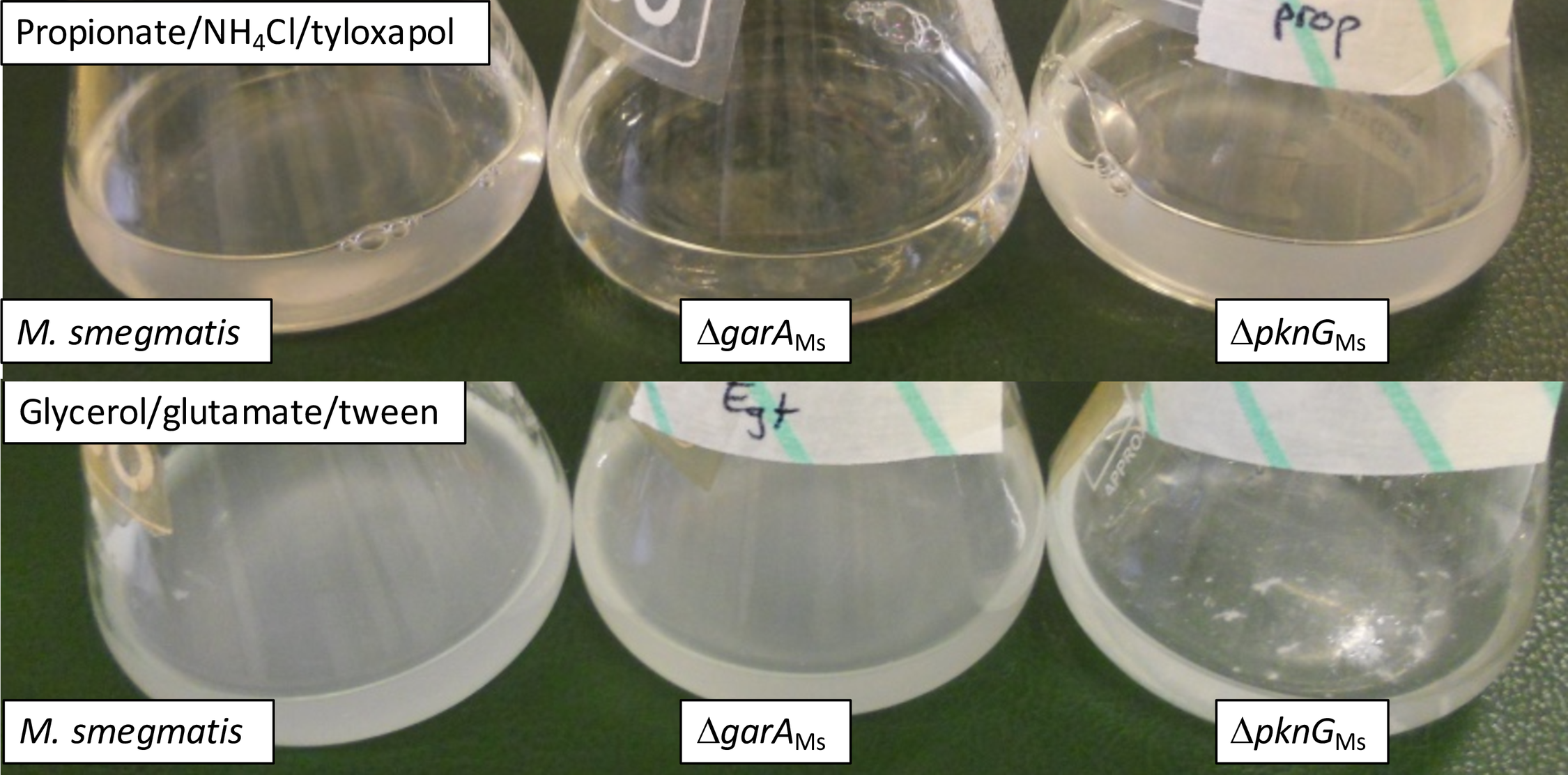

Supplement: S5 Fig — M. smegmatis garA mutant grew poorly on propionate, while M. smegmatis pknG mutant grew poorly and formed clumps when glutamate was the sole nitrogen source. (TIFF) [file ppat.1006399.s014.tiff]

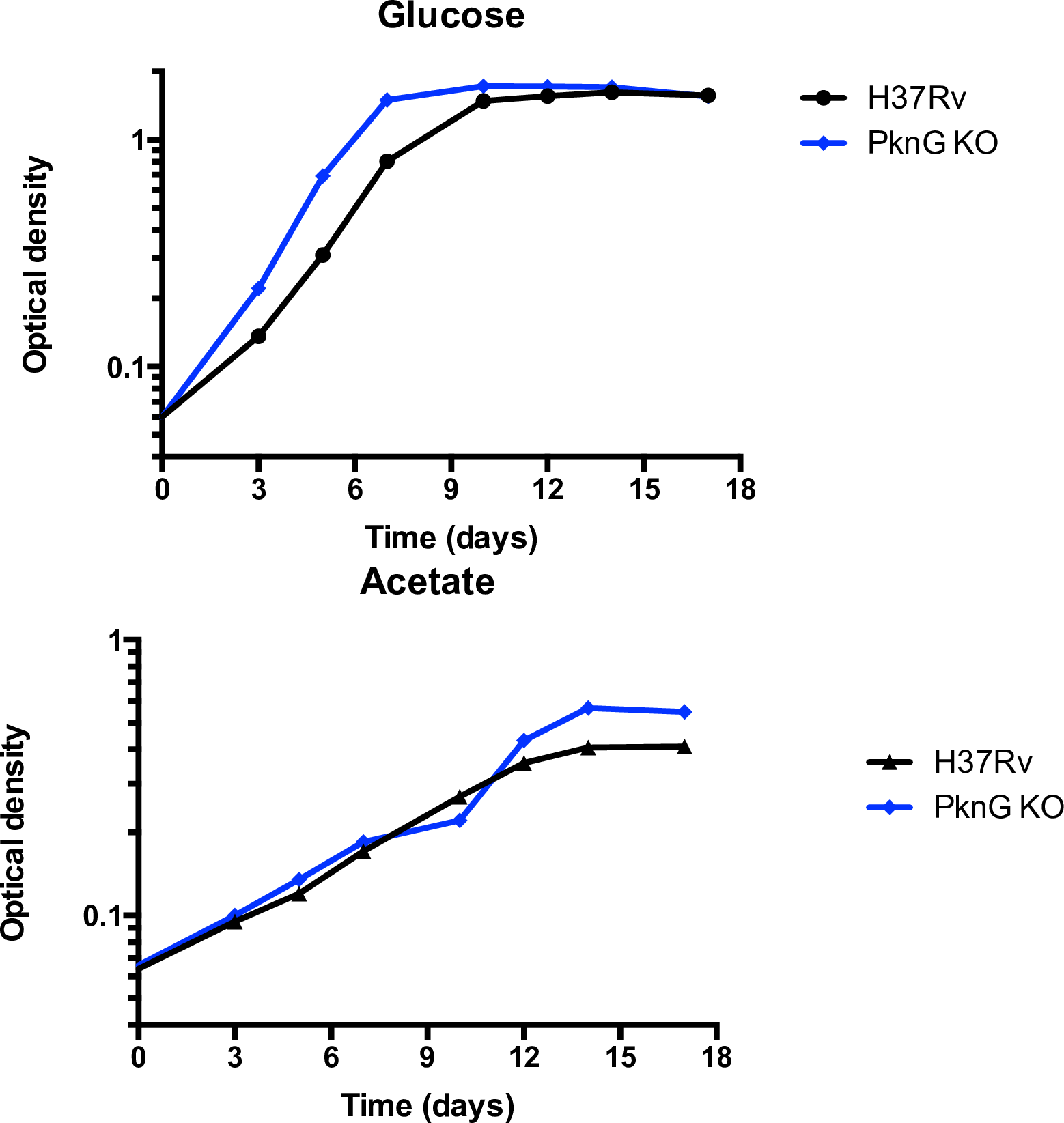

Supplement: S6 Fig — Alternative carbon sources were tested to characterise the nutrient-dependent growth defect of ΔpknGMt. The pknG deficient strain had a growth defect only when glutamate or asparagine were the sole carbon source in minimal medium (main manuscript Fig 3). When other carbon sources were used of ΔpknGMt (blue diamonds) grew at the same rate as the parent strain (black circles). Glucose or acetate were added to minimal Sauton’s medium at 0.2%. Graphs show measurements from a single experiment that is representative of multiple independent experiments. (TIFF) [file ppat.1006399.s015.tiff]

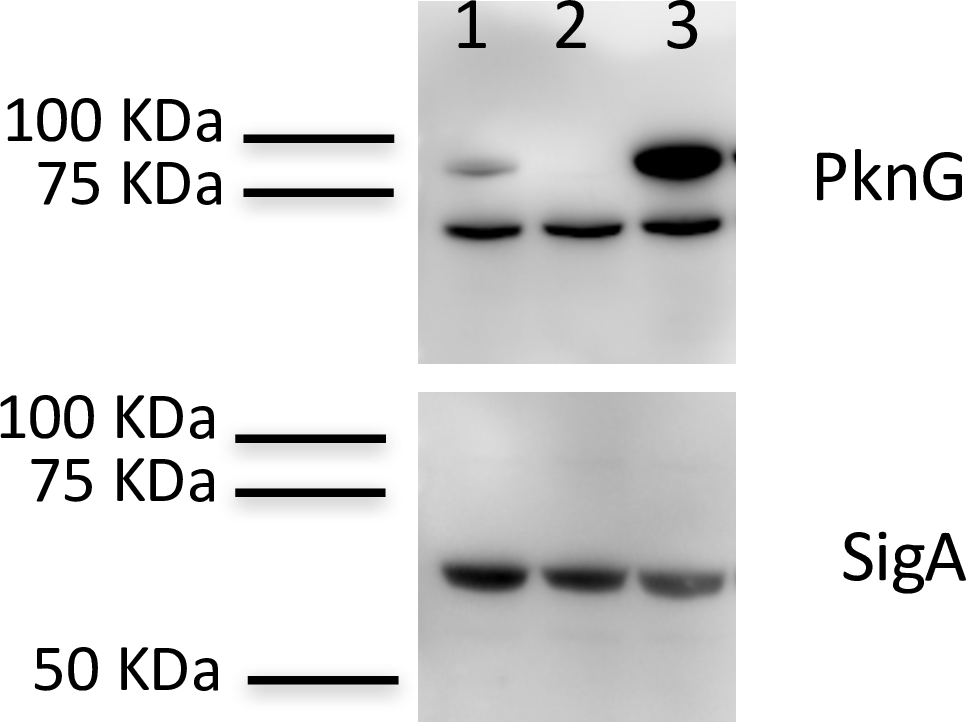

Supplement: S7 Fig — M. tuberculosis H37Rv (1), ΔpknGMt (2) and ΔpknGMt + pAL299 (3) whole cell lysates were analysed by western blotting. Equal amount of cell equivalents were loaded and proteins were detected using anti-PknG serum diluted 1:4000, anti SigA serum diluted 1:4000 and HRP-anti rabbit antibody. (TIFF) [file ppat.1006399.s016.tiff]

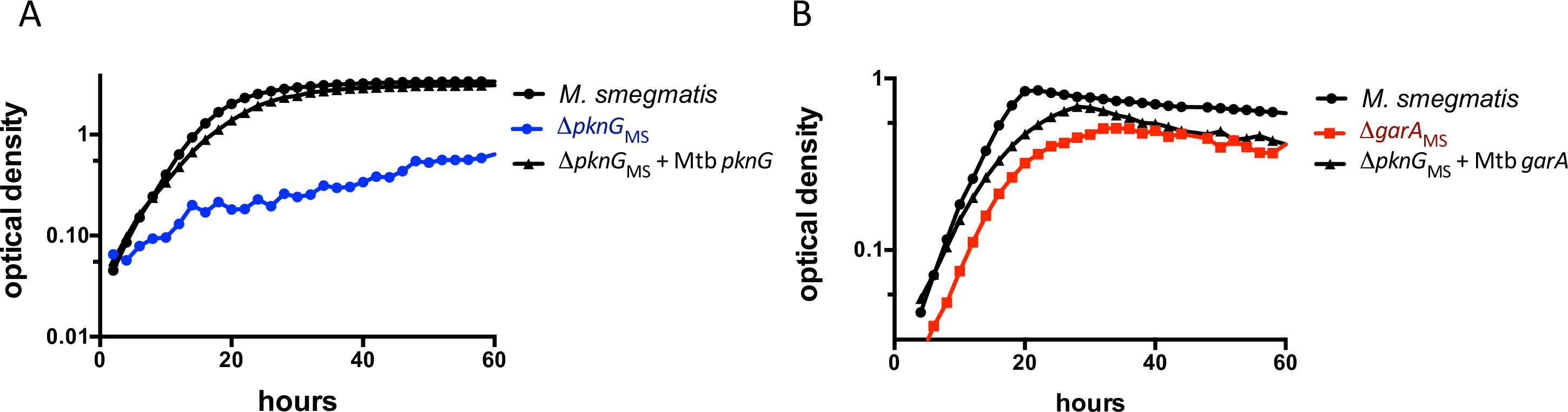

Supplement: S8 Fig — (A) M. tuberculosis pknG was introduced into ΔpknGMs leading to restoration of the ability to grow on minimal Sauton’s medium containing 10 mM glutamate, 1% glycerol and 0.05% tween 80 as sole carbon/nitrogen sources. (B) M. tuberculosis garA was introduced into ΔgarAMs leading to partial restoration of the ability to grow on minimal Sauton’s medium containing 20 mM propionate and 10 mM NH4Cl as sole carbon/nitrogen source with 0.05% tyloxapol to prevent clumping. The growth experiment shown is a representative of three independent experiments. Data plotted are mean and standard deviation of three technical replicates and are representative of 3 independent experiments. (TIFF) [file ppat.1006399.s017.tiff]

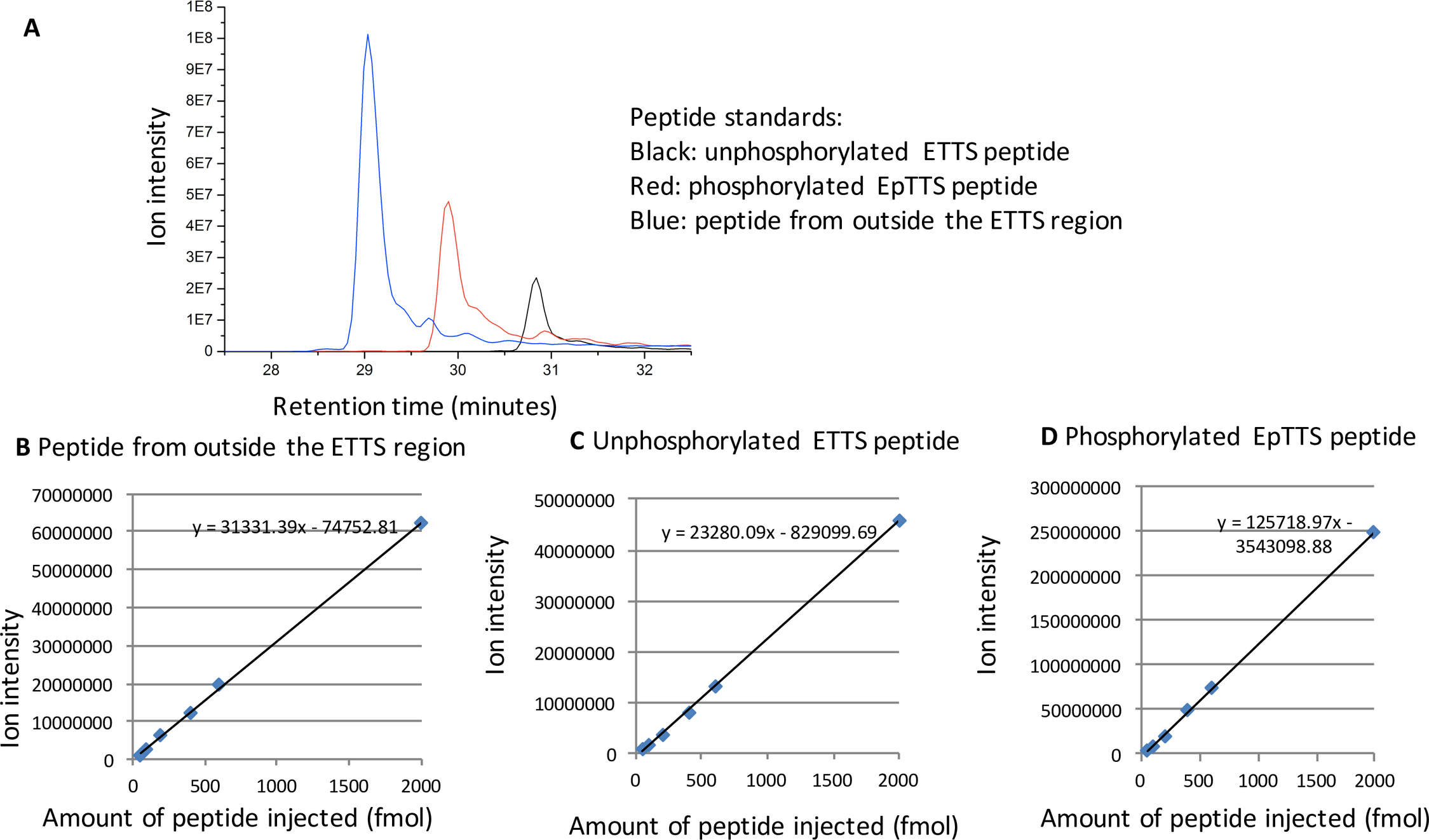

Supplement: S9 Fig — In preparation for analysis of tryptic peptides of GarA (main Fig 4), equivalent synthetic peptides were analysed by LC-MS/MS as a mix in 1:1:1 molar ratio (A) or singly at a range of concentrations (B-D). (TIFF) [file ppat.1006399.s018.tiff]

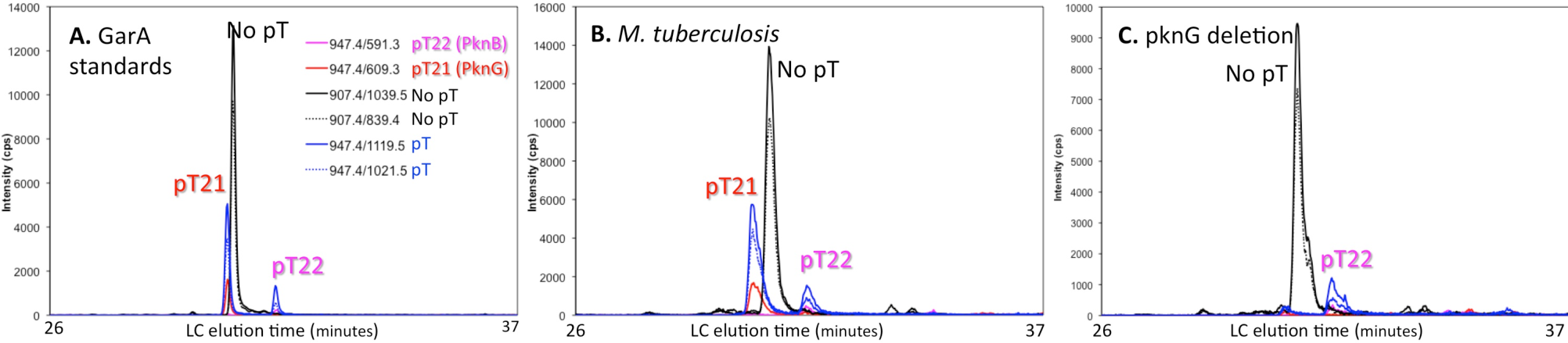

Supplement: S10 Fig — The tryptic peptide corresponding to T21-phosphorylated GarA (EpTTS) was absent from extracts of ΔpknGMt suggesting that PknG is the main kinase responsible for phosphorylating GarA. (TIFF) [file ppat.1006399.s019.tiff]

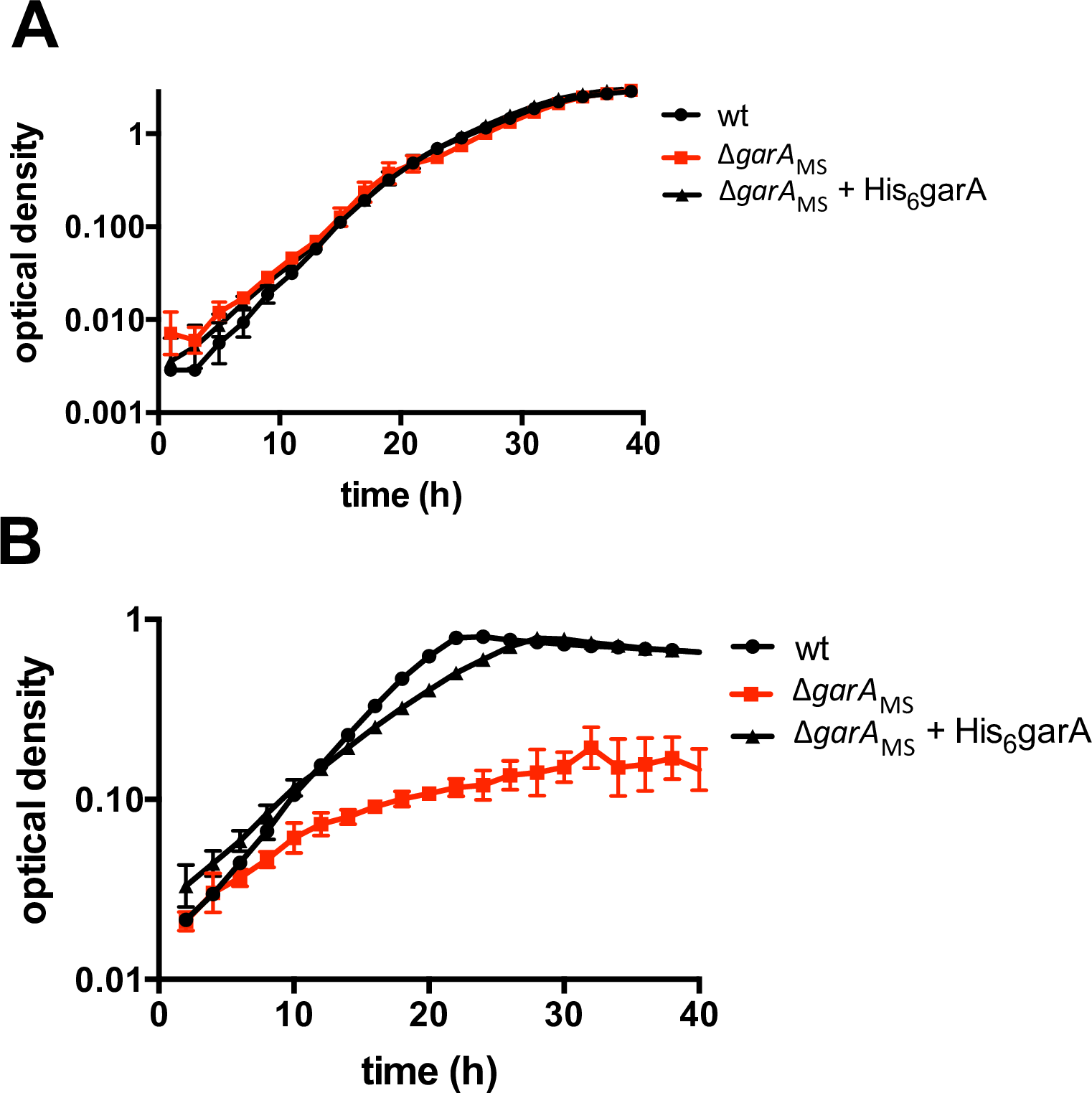

Supplement: S11 Fig — Hexahistidine-tagged GarA can complement the growth defect of ΔgarAMs. Growth curves of M. smegmatis mc2155, ΔgarAMs, and complemented ΔgarAMs strain with His6 tag in standard Sauton’s medium (A) or modified Sauton’s (B) containing 20 mM propionate, tyloxapol and 10 mM NH4Cl. The growth experiment shown is a representative of three independent experiments. Data plotted are mean and standard deviation of five technical replicates. (TIFF) [file ppat.1006399.s020.tiff]

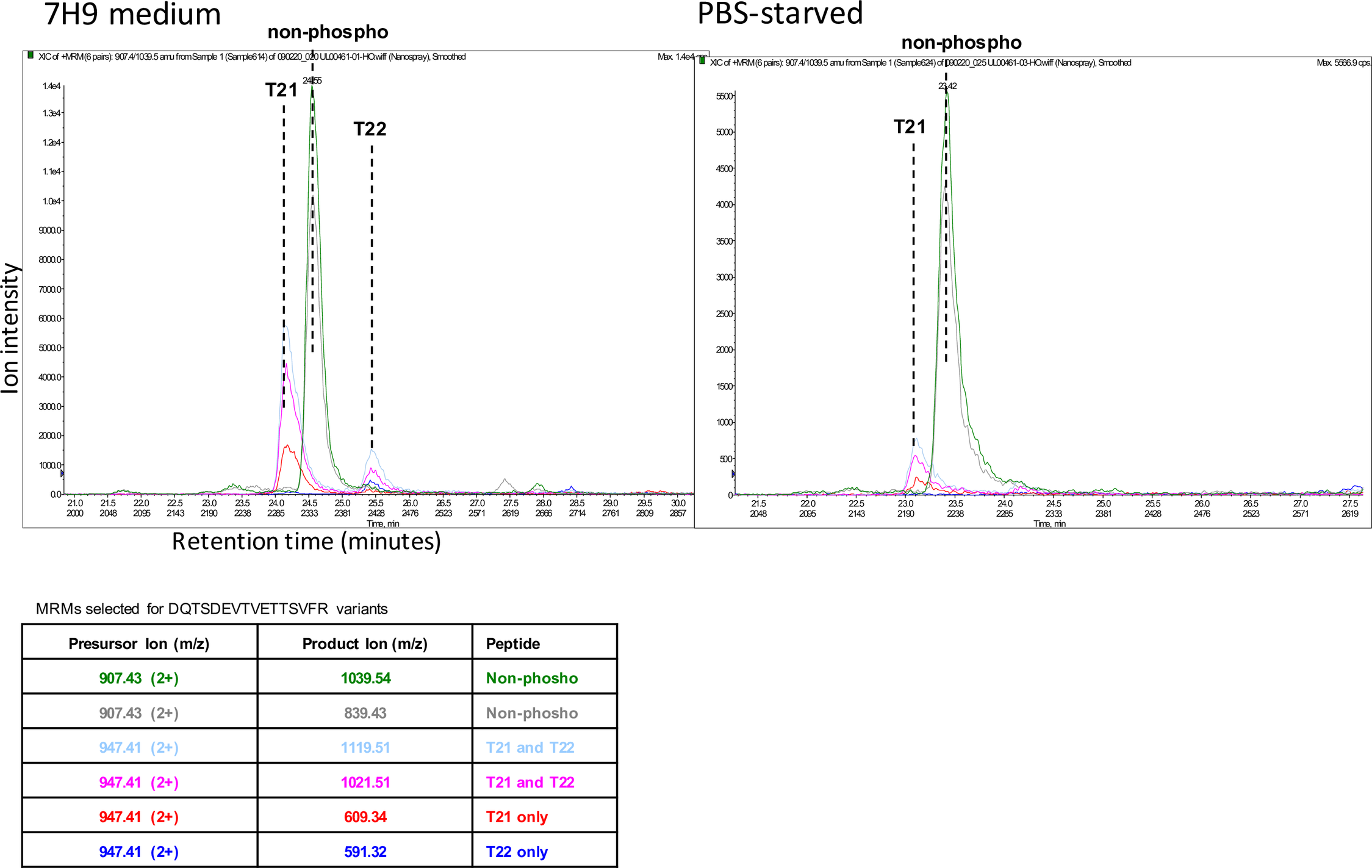

Supplement: S12 Fig — Graphs are representative of at least 3 independent experiments. (TIFF) [file ppat.1006399.s021.tiff]

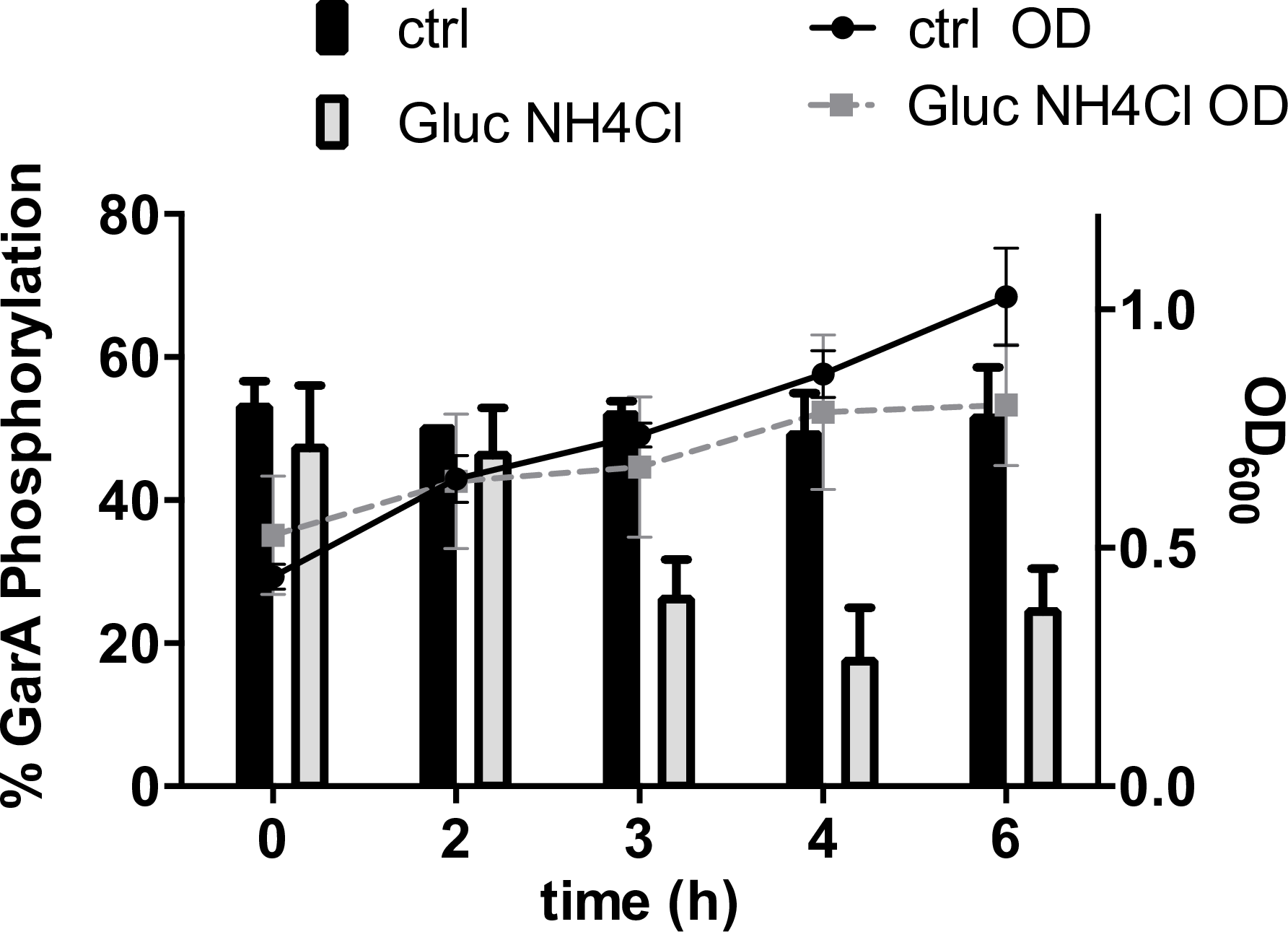

Supplement: S13 Fig — To verify that loss of GarA phosphorylation was related to change of nutrients rather than cessation of growth, M. smegmatis was maintained in phosphorylation medium (black bars show % of GarA phosphorylated and black line shows optical density) or switched to minimal medium at t = 0 (grey bars show % phosphorylation and grey line shows optical density) and optical density was monitored in parallel to sampling for phosphorylation analysis. (TIFF) [file ppat.1006399.s022.tiff]

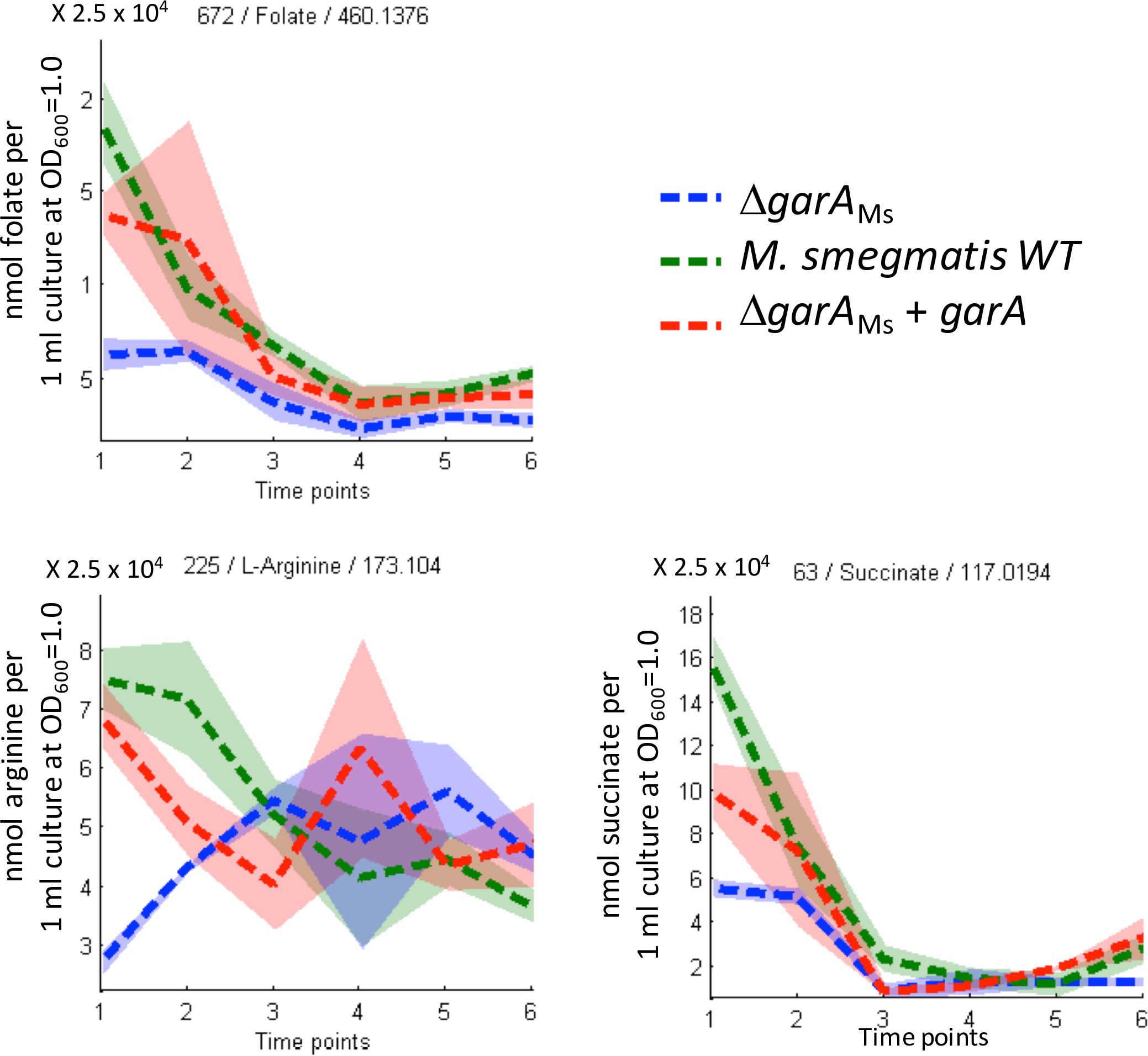

Supplement: S14 Fig — These measurements verify that the ΔgarAMs cells had not lysed at day 28 (time point 6). The line represents the mean and shaded area shows standard deviation from 3 independent cultures. (TIFF) [file ppat.1006399.s023.tiff]

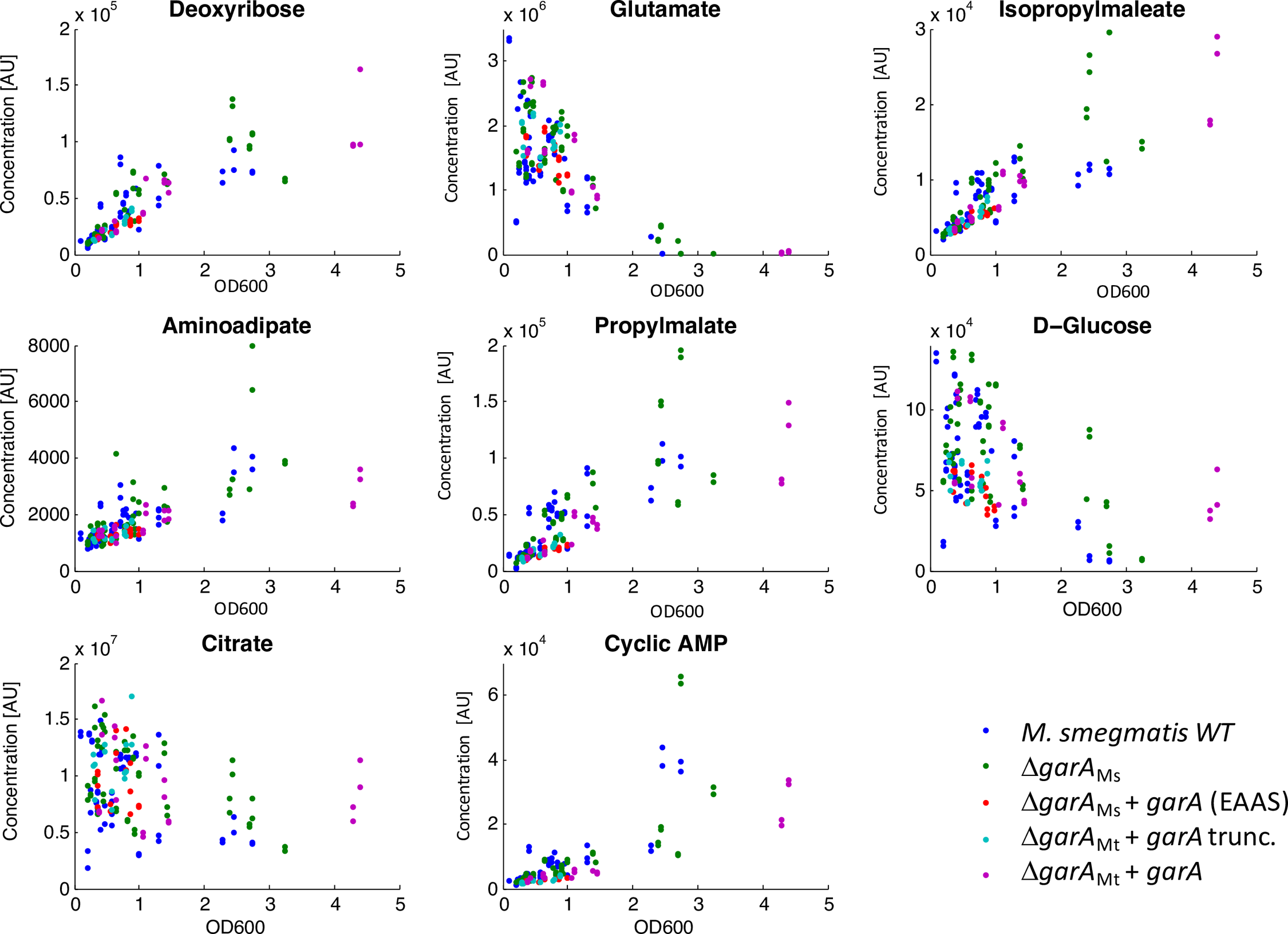

Supplement: S15 Fig — Extracellular metabolite concentrations of M. smegmatis mutant strains grown in Middlebrook 7H9 broth did not show significant changes compared to wild type. (TIFF) [file ppat.1006399.s024.tiff]

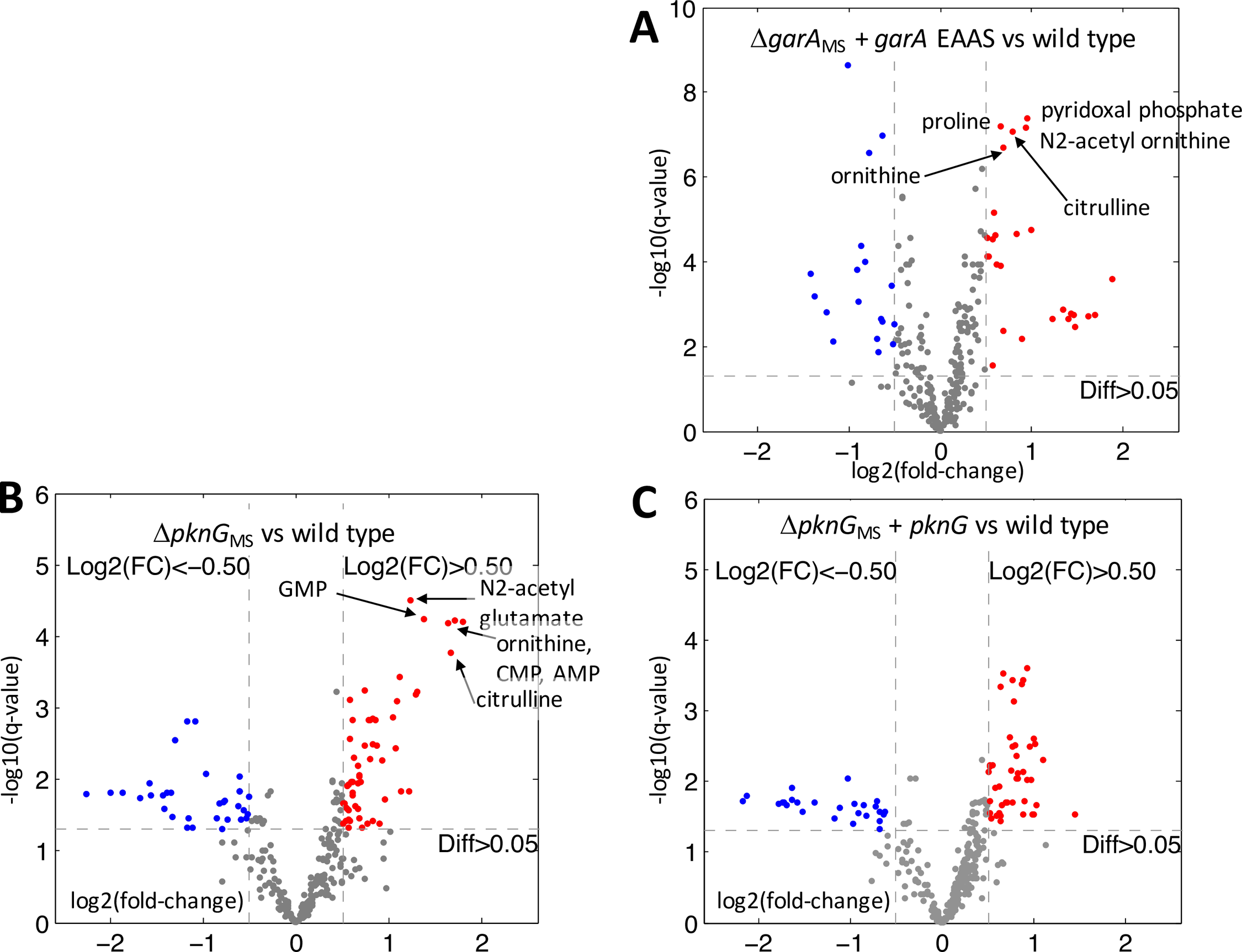

Supplement: S16 Fig — (A) M. smegmatis expressing unphosphorylatable GarA had altered intracellular metabolites compared to wild type. The changes were similar to those seen in the strain expressing truncated GarA (main text Fig 7C). (B) ΔpknGMs had an altered intracellular metabolome compared to wild type. (C) reintroduction of pknG partially restored the perturbations. All strains were grown in Middlebrook 7H9 broth. (TIFF) [file ppat.1006399.s025.tiff]

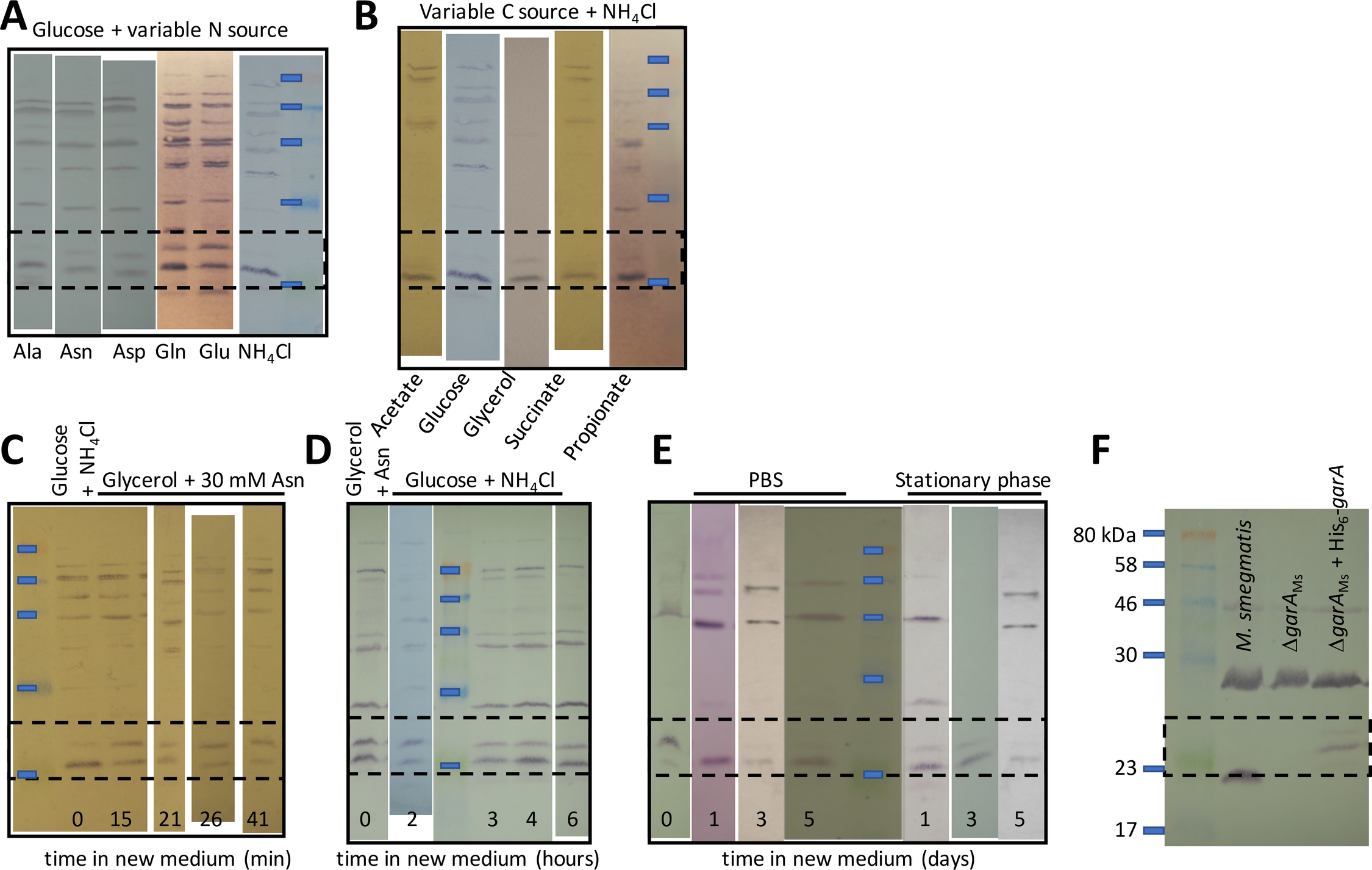

Supplement: S17 Fig — Dashed lines mark the regions of the blots displayed in Fig 5. These images are representative of at least 3 samples for each condition that were analysed to calculate the ratios in Fig 5. The anti-GarA serum gave several non-specific bands, but His6-GarA was identified with confidence by comparison with molecular weight markers and by comparison with garA-deleted M. smegmatis (panel F). (TIFF) [file ppat.1006399.s026.tiff]
